# A conceptual model of the impact of including carers in museum programmes for people with dementia

**DOI:** 10.1177/14713012221126803

**Published:** 2022-09-22

**Authors:** Debbie Kinsey, Noreen Orr, Rob Anderson, Iain Lang

**Affiliations:** 3286 The University of Exeter Medical School, Exeter, UK

**Keywords:** realist evaluation, dementias, caregiving, museums, arts interventions

## Abstract

**Background:**

Research has highlighted a need for more theoretical work in arts interventions, including the role of the dyad. This study aimed to test theories from a literature review on the impact of including carers in museum programmes for people with dementia, and develop a model which can be used in other programmes to consider the impact of including carers more broadly.

**Methods:**

Using a realist evaluation approach, theory was developed through interviews and participant-observation at six museums in the UK.

**Findings:**

The impacts of including carers fell into seven broad areas – caring responsibility, session function, controlling access, preventing engagement, comparisons and losses, long-term impact of in-the-moment activities, and reducing social isolation and opening up the museum.

**Conclusions:**

Including carers may have both unanticipated benefits and negative consequences, and greater attention is needed on how both carers and people with dementia can be supported in shared sessions. Carers should be viewed as participants of programmes, and can even be the main beneficiaries, even where the programme is ostensibly ‘for’ the person with dementia – it’s not simply that carers are the enablers of, or barriers to, the impacts on the person with dementia.

## Introduction

Dementia can have a relational impact affecting both the person with dementia and those around them, as well as the relationship between them (e.g. Moon & Adams, 2013; Wadham et al., 2016). The formal support within health and social care available to carers and people with dementia can be limited, under-funded, and difficult to navigate (Department of Health and Social Care, 2018). Common means of support such as respite are underutilised, perhaps because of a misalignment between carer and policy conceptualisations of respite (O′Shea et al., 2019). Many people therefore receive support through the third sector (Dodd et al., 2014). Dyadic cultural arts interventions are increasingly being offered to people with dementia and their carers, with the aim of providing a meaningful, shared experience, linked to maintenance of individual and relationship identity (e.g. De Medeiros & Basting, 2014; Mittelman & Epstein, 2009). Museums are one venue providing dyadic cultural arts interventions, including a particular type of non-reminiscence-based programme focussed on a shared, enjoyable experience (Mittelman & Epstein, 2009).

Studies evaluating museum programmes have found a number of positive impacts, such as social enjoyment (e.g. MacPherson, et al, 2009), improved mood (e.g. Eekelaar, et al., 2012), and stress relief (e.g. Lamar & Luke, 2016). Two studies examining *how* these programmes have an impact both developed models which describe positive individual and relational impacts, within the valued space of the museum (Burnside et al., 2017; Camic et al., 2016).

While most studies evaluating museum programmes focus on positive outcomes, two reported negative outcomes related to the inclusion of carers. MacPherson et al (2009) found professional carers created ‘excess disability’ in the participants with dementia in that the carers did tasks for the person they were able to do themselves once the carers left. Windle et al. (2018) made the participation of carers optional, and stated that the presence of carers occasionally needed careful management by facilitators.

The museum-based research does not consider whether there may also be potential negative impacts on carers themselves. Research on other interventions has suggested there can be a negative impact of dyadic interventions on the carer, such as increased stress (e.g. Melunsky et al., 2015; Woods et al., 2016).

Studies of cultural arts programmes in general have also been criticised for a focus on positive outcomes without acknowledging potential negative effects (Daykin et al., 2020). Much of the research on museum programmes has also focused on family, rather than professional, carers, or not differentiated between dyad types. This may be important given the different socio-relational challenges faced by different dyad types (Rausch et al., 2017).

There is a need for more theoretically-informed work which considers the role of the dyad specifically in arts interventions (Bourne et al., 2020). Kinsey et al.’s (2019) realist review developed 16 theory statements on the impact of including carers in museum programmes within four themes of outcomes: seeing the person with dementia in a new way and building relationships, shared respite, excess disability, and reduced social isolation. The review suggested including carers can have both positive and negative impacts on the carer, the person with dementia, and the relationship between them. For example, one theory statement suggested that, where the dyad’s home interactions are mainly around caring, enabling facilitation in a nonmedical setting in which the carer has no caring responsibilities means the dyad can enjoy the activity together on an equal basis, which leads to the dyad experiencing shared respite from dementia, which also helps to strengthen their relationship. Whereas another suggested some carers compare the person with dementia’s current abilities to their past abilities, or negatively with others in the group, which can lead to reinforcing limitations or highlighting losses. However, the theories were limited by the scope of the current literature, and highlighted more research considering dyadic context was needed to understand who may, and may not, benefit from dyadic museum programmes.

This aligns with research on other types of dyadic interventions, which suggests they may not benefit all. For example, there may be a negative impact of interventions promoting closeness when what the carer needs is to detach emotionally to protect their own mental health (Fauth et al., 2012; Rapaport et al, 2017). However, it contrasts with Bourne et al’s (2020) review of psychosocial outcomes of dyadic arts interventions, which found no reports of negative effects of dyadic visual arts interventions. Kinsey et al (2019) specifically investigated processes alongside outcomes, whereas Bourne et al (2020) investigated outcomes more generally, which may account for the contrasting findings.

As most programmes include carers, but research suggests dyadic interventions may not benefit all, it is important to consider the impact of including carers, particularly as previous reviews on museums contrast. A focus on underlying processes and mechanisms may not only strengthen the research base for cultural arts programmes in dementia (De Medeiros & Basting, 2014), but also support broader implementation through understanding what works in different settings and for different people (Windle et al., 2018).

Our aim was to empirically test and refine theories on the impact of including carers in museum programmes for people with dementia, developed through Kinsey et al.’s (2019) review. The refined theories are then used to develop a model which can be used and tested in other kinds of programmes to consider the impact of including carers more broadly.

## Methods

This study used a realist evaluation approach, building on a realist synthesis (Kinsey et al., 2019). Realist methodology does not prescribe a particular set of methods in evaluation but seeks to open the ‘black box’ of the intervention to understand causal processes, understood as the interaction between aspects of context and underlying mechanisms, leading to outcomes (Pawson & Tilley, 1997). The methods should therefore aim to investigate participants’ reasoning and responses, as well as contexts and outcomes.

### Study design

The study used two means of qualitative data collection across six different sites:

1. Participant-observation within museum sessions. The level of involvement in sessions was decided collaboratively with staff to suit the needs of each individual programme. It usually involved helping with set-up and being involved with activities with the attendees.
2. Interviews with session attendees (people with dementia and carers) and museum staff. Dyads could choose to have individual or joint interviews, and staff were interviewed individually. Interviews used a realist technique intended to explore the experiences within sessions; how these relate to the review theories; and individuals’ explanations of ideas and actions which could provide insight into their reasoning (and so theory mechanisms) (Manzano, 2016).

### Study sites

Six programmes for people with dementia and their carers from were recruited purposively to include a range of museum types (e.g. primarily art- or history-based), sizes, and locations. Included museums were based in South-West England, the Midlands, and London, and ranged in size from an average of 250,000 visits a year to an average of four million a year. The programmes ranged from established for several years to having only run for a few months.

### Participants

Session attendees were eligible for inclusion if:

• One of the dyad self-described as having a diagnosis of dementia;
• They both attended the programme.

They were excluded if:

• One or both people lacked capacity to consent;
• One of the dyad did not attend the programme (i.e. if a person with dementia attended with a paid carer and not their spouse, the person with dementia and their paid carer are the dyad eligible for inclusion, not the person with dementia and their spouse);
• The professional carer was paid for by the NHS or social care providers due to ethical approval restrictions.

Museum staff were eligible for inclusion if they participated in the development or delivery of the programme.

### Numbers at each site

Table 1 details the number of sessions attended, sessions included, and number of interviews at each site.

**Table 1.** Number of sessions attended and participants included at each site.

	Session observations		Interviews		
	Number of sessions attended	Number of sessions in which fieldnotes possible	People with dementia	Carers	Staff
Site 1	6	5	0	0	2
Site 2	4	4 (staff meetings only)	0	0	2
Site 3	4	2	0	0	1
Site 4	6	3	2	2	2
Site 5	5	3	0	0	1
Site 6	3	2	0	0	4
TOTAL	28	19	2	2	12

### Analysis

The analysis was inductive (creating theory from the data), deductive (testing theory with the data), and retroductive (inferring or identifying underlying causal mechanisms, which includes inductive, deductive, and abductive thinking; Sayer, 2000). It used a combination of connecting and categorising strategies so that the connecting analysis considered the context and connections that may not have been included in the categorising analyses (Maxwell, 2012). Data were coded for contribution to theory and the impacts of carers. Cross-data comparisons were made of coding, both within the same data type (e.g. comparing interviews) and between different data types (e.g. between interviews and fieldnotes). Cross-data comparisons were also made between research sites. This was not a case study approach but was to ensure how the data were examined considered the overarching context of each programme. This enabled consideration of factors not apparent in the data itself, such as museum size or attendee-recruitment method.

‘Dyad analysis’ was also conducted wherever possible. Where a dyad appeared in more than one transcript (such as two or more session fieldnotes), extracts from those transcripts were brought together and coded for how they were using the programme, for example whether they were doing activities together or separately, or whether museum staff took on a caring role. Narrative summaries were created for each dyad on how they used the programme, including contextual factors related to caring such as whether one of the dyad needed support to walk.

This realist framework was used to refine, refute, and create new theory in relation to the research questions and was an iterative process throughout the study.

We then listed key concepts of the programme theories and cased similar ideas (Ragin & Becker, 1992) to develop the conceptual model of core processes.

## Findings

The analysis resulted in 28 evidence-informed programme theories (listed in Supplementary File 1; Supplementary File 2 details the process of revising the review theories, with supporting evidence), which were then developed into a conceptual model of the processes involved when carers are included in these types of programmes. They include both positive and negative impacts on the person with dementia, the carer, and the relationship between them. A mapping of the core processes to the programme theories is given in Supplementary File 3. These core processes do not stand alone, but sit within the context of how these museum programmes work – equal participation, in-the-moment wellbeing, not being about dementia, enabling facilitation, and held in a community-based, valued place.

**Figure 1.**
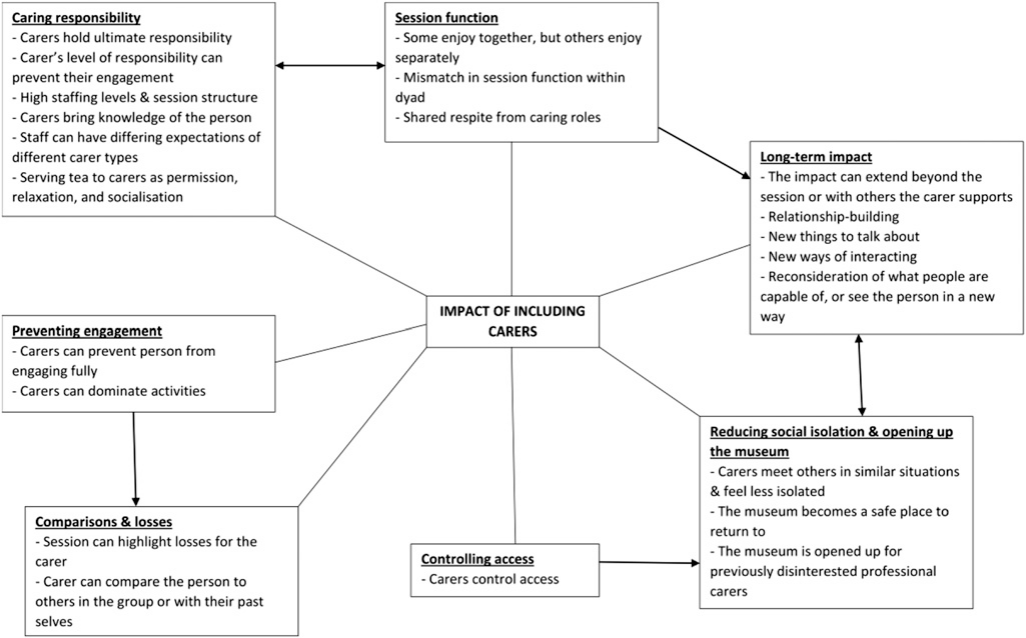
Conceptual model of the impact of including carers.

Figure 1 shows the final model, which suggests the impacts of carers fall into seven broad areas – caring responsibility, session function, controlling access, preventing engagement, comparisons and losses, long-term impact of in-the-moment activities, and reducing social isolation and opening up the museum. This findings section will explain each of these broad areas, and the connected core processes, to present the conceptual platform of how including carers in museum programmes can have an impact.

There are many different kinds of carers, including spouses, friends, children, care home workers, and community respite workers. As different kinds of caring dyads may bring differing contexts and dynamics, the term ‘professional carer’ will be used to describe all types of paid carers (where caring is their paid job), and the term ‘family carer’ will be used to describe ‘informal’ carers such as spouses, children, and friends. Where the carer type could not be sufficiently differentiated, or the finding refers to all carer types, the standalone term ‘carer’ will be used.

## Caring responsibilities

Core processes:

a. Carers hold the ultimate responsibility for the person with dementia (so the museum does not have to).
b. The carer’s level of responsibilities in the group can prevent them from engaging in and enjoying the activity.
c. High staffing levels and the session structure take some responsibilities from the carer.
d. Serving tea to carers helps them to feel the session is also for them, to socialise, and to relax after a stressful journey.
e. The carer can enable the person with dementia to participate.
f. Carers bring knowledge of the person with dementia which helps the museum staff to support the person with dementia.
g. Museum staff can have differing expectations of different carer types, impacting their experiences.

‘Caring responsibility’ included supporting the person with dementia physically or emotionally, giving staff information about their needs, and being overall responsible for the safety or wellbeing of the person with dementia. Safeguarding was described by staff as the main reason for including carers, whether the programme initially planned to include them or not. Although staff described limits to the support they were able to provide, all programmes took some responsibility from the carer, due to structured nature of sessions which took care of the content and provided some support for the process. The higher staffing levels in these sessions also helped minimise what carers needed to do, to an extent.

All but site 3 included tea during the session. Tea being made and served by staff was a way of modelling to the carer that the session was also for them, they could relax and enjoy it, and that they did not hold all the responsibility. Several carers were surprised when they did not have to make the tea themselves. It was also a way of supporting carers by allowing space to de-stress after the journey.

*[Tea and biscuits] sets the tone, you know, that this is going to be fun or it’s relaxed and you’re welcome here*.

– *Carer interview, site 4*

Programmes were generally seen by staff as being primarily for the person with dementia but also for the carer to enjoy, with efforts made to ensure the carer knew they were included. However, some staff suggested the sessions were more for family carers to enjoy than professional carers, though this varied between staff members even at the same site.

*Well, if they’re paid, it’s very different to if they’re not paid, I think*.

*– Staff interview, site 4*

## Session function

Core processes:

h. Some dyads enjoy the session together, but others use it as an opportunity for traditional respite from one another.
i. The dyad can experience shared respite from dementia and their caring roles.
j. There can be mismatch in the way the carer and the person with dementia wants to use the session, which can cause tension or distress

One of the common aims of these programmes is an ‘enjoying together’ for the dyad, and one of the key positive outcomes outlined in the review (Kinsey et al., 2019) was the idea of shared respite – a break, together, away from dementia and their roles as carer and cared-for, which also appeared to be the case for some dyads in this study. However, it was also apparent dyads use the session in different ways. Whilst some enjoy the session together, for others the aim is not ‘enjoying together’ but enjoying separately, much like a more traditional model of respite.

*During tea, [the dyad] sat at the same part of the table, but each on one side of the table’s corner and mostly talked to other people in the group or staff. During the session they rarely walked together between objects and never sat together. They talked to other people in the group but rarely to each other*.

– *Fieldnotes, site 6*

At times there was a mismatch in the way the person with dementia and carer wanted to use the session, with one person wanting closeness and the other to be separate, resulting in one person feeling upset or frustrated. Session function could also change as the person with dementia’s condition progressed or they needed greater support from their carer. This transition period, when a carer who uses the session for more traditional respite but then must increasingly be with the person with dementia to support them, may be challenging for some. For example, one dyad (Robert, carer, and Stella, person with dementia, spouses) initially spent the sessions separately, with Robert using it to have time to himself to enjoy the museum. During the research, Stella’s support needs gradually increased and Robert increasingly needed to support Stella, and he often expressed frustration and immediately moved away to be on his own after helping her.

This interaction of caring responsibilities with the function of sessions for the dyad as a whole, or for them as individuals, is complex. Some carers appeared to enjoy the session and feel respite at the same time as having several caring responsibilities, whereas others needed to have few, to no, responsibilities for enjoyment or respite.

For example, in contrast to Robert and Stella, despite Lucy (carer, wife) having to be with George (person with dementia, husband) at all times due to his support needs, the sessions appeared to be more for her to enjoy as George was usually not engaged with them. They seemed to be a form of respite for Lucy through the activities themselves and having conversations with others.

## Controlling access

Core process:

k. Carers control whether the dyad (and so person with dementia) attends and/or returns.

Related to the ways carers supported the person with dementia in the session if they needed it, carers also enabled the person to attend the session through finding out about it, booking, and dealing with the logistics of travel.

As the carer is the one who found out about the session and booked, they effectively controlled whether the dyad (and so the person with dementia) attended. The site 5 manager talked at length in her interview about how much research and planning went into making sessions dementia-friendly, including talking to carers about how to adapt activities for people with dementia’s needs. During this research, these sessions were poorly attended, with usually only a few participants or cancelled due to lack of bookings. At our final session at site 5, the manager said she recently delivered an outreach session to carers in which she discussed the tours, and demonstrated example activities. She said the carers told her they would not have attended the tours prior to the outreach session as they had never visited site 5 and were not sure how suitable the activities or setting would be. The bookings for tours following the outreach did then increase. This suggests programmes also need to focus on what makes it accessible or comfortable for carers, not just people with dementia, even where the session is primarily for the person with dementia.

## Preventing engagement

Core processes:

l. Carers can prevent the person with dementia from engaging fully.
m. Carers can dominate the activities or discussions.

These core processes were derived from theories which included the carer trying to make the person with dementia engage in a way they do not want to, carers intervening unnecessarily, and carers dominating group discussions.

One part of the ethos of these programmes, is the idea of meeting the attendee ‘in the moment’ – not who they were in the past or what they were interested in then, but with interaction based on who they are and what they are interested in on the day of the museum visit. There were occasions where some carers struggled with this and tried to prompt particular memories linked to the activity or discussion from the person with dementia.

There were also times the carer tried to make the person with dementia engage in a particular way with the activity or discussion, based on things they used to enjoy or an aspect of their identity. For example, at site 4, a person with dementia who used to be a musician did not want to get actively involved in a music-making activity and said he just wanted to listen. His wife had mentioned earlier that they had sold his instruments as he was no longer able to play them – so the person with dementia may not have wanted to get involved due to grief for that skill or because he was no longer interested. However, the carer persisted in trying to get him to make music, and later to answer music-related questions. The person with dementia became increasingly annoyed with his wife during the session.

In most sessions, general questions to the group were answered by carers, not people with dementia. Staff interviews suggested the importance of facilitation in whether, and how, carers or people with dementia were involved in group conversations, but also that the session being a new, enjoyable activity for the carer, which they do not have very often, meant carers could dominate conversations through enthusiasm.

*I think one of the dangers sometimes then is [the carers] get really comfortable and also really enjoy it and sometimes they dominate just by the amount of time that they then want to speak, because it’s also a space that they’re not getting very much*.

- *Staff interview, site 1*

Sometimes carers could prevent the person with dementia engaging if they were worried about the person, or anxious about the museum space and the correct ‘etiquette’, and so spoke on their behalf or did tasks for them they could actually manage themselves.

## Comparisons & losses

Core processes:

n. The session can highlight losses for the family carer.
o. The family carer can compare the person with dementia to other people with dementia in the group or with their past selves.

These core processes relate to the carer forcing the person with dementia to engage in a particular way that is more consistent with their past self, the carer comparing the person’s current abilities to their past abilities, and losses being highlighted for the carer. This section relates specifically to family carers, as these issues are more likely to apply to them due to usually having longer relationships, and more emotional history, with the person they support.

Some carers found it upsetting when the person with dementia did not have the same ability as they had previously. Sometimes this was because the activity was outside their usual routine, which meant they were asked to do things they may have stopped doing in their day-to-day life and the carer had not realised:

*There was [wife] and [husband] who came today. The first session they came to, we asked them to write some, write their name on something, and [husband] couldn’t write his name and [wife] didn’t know he couldn’t write. And I think that really upset her. Because she didn’t know, so that was quite sad for her*.

- *Staff interview, site 4*

Sometimes carers compared the person they supported with other people with dementia in the group who had greater difficulties and this highlighted a possible negative future:

*Well the only, yeah the bad thing [about the programme] would be seeing somebody gradually decline and seeing the difficulty they have*.

- *Carer interview, site 4*

Although respite was generally a positive outcome, for some carers feeling respite from their caring role could also highlight losses for them. A family carer being outside of their caring role, which they may not have felt for some time, makes them realise there are activities they miss which they used to enjoy, highlighting the carer’s own losses. However, could become a positive when the carer then has the resources to seek out other activities that alleviate some of that loss.

*The wife had been really creative and she actually got really emotional in the session because I think she just realised how she didn’t have creativity in her life anymore and that, that reminded her of that. And that, she then got in touch with a craft group near [city]. She managed to get that as part of her regular week, to have something else going on*.

- *Staff interview, site 1*

## Long-term impact of in-the-moment activities

Core processes:

p. The impact of the programme can extend beyond the session or with other people with dementia who did not attend.
q. The session can build the relationship between the carer and the person with dementia.
r. The session gives the dyad new things to talk about outside of caring tasks.
s. Carers can learn new ways of interacting with the person with dementia and/or new strategies for working with them.
t. The carer may reconsider what the person with dementia (or people with dementia generally) are capable of doing, or see the person with dementia in a new way.

This evaluation did not include a long-term follow-up of dyads outside of the sessions to assess any long-term impacts. However, there were three potential long-term impacts of including carers suggested by the data: seeking out or replicating the activity, having new things to talk about, and relationship-building.

Carers from care homes were described as intending to replicate the art-making activities in the care home with other residents, and family carers were described as seeking out similar activities. By including carers, who, as previously discussed, were the ones who found and booked activities for the dyad, the person with dementia (and the carer) could then get involved in similar activities elsewhere, or care home residents who did not attend the session could enjoy a similar activity in the home. However, it can only be suggested as an intention, as there was no long-term follow-up to determine if they did re-create the activities afterwards.

Attending the session together gave some dyads new things to discuss outside the session and new things to share with others. If the person with dementia attended the session alone, they may have had new things to discuss with the carer when they returned home. However, one dyad suggested it helped it was a shared experience, as they could prompt each other about the experience. It may also link with the idea of shared respite from caring roles, as it is a source of conversation unrelated to caring tasks.

*Person with dementia: … I actually very much enjoy listening to it, but if you ask me questions afterwards then I wouldn’t be able to deal with that. You can understand that*.

*Carer: But then you always remember if I remind you of things, you do normally remember us doing it in a session*.

– *Professional carer & person with dementia interview, site 4*

Some carers shifted their expectations of what the person with dementia would be able to do or how they would engage with activities. However, one staff member suggested it is not about professional carers shifting their thinking in terms of what the *individual* person with dementia is capable of, but it is more that their thinking shifts about what is possible in terms of activities with people with dementia more generally, for example the type and complexity of activities people can engage in and enjoy.

Some dyads learnt new things about each other and shared an enjoyable experience, which built their relationship with one another.

*And [at] our last [site] tour, the paid carer said “oh it was really nice to see, to actually sing with him and to get to know him more as well”*.

- *Staff interview, site 5*

However, some dyads said they had not learnt new things about each other due to their longstanding relationship. It may be that carers are more likely to learn something new about the person with dementia when they do not already know them well, for example, professional carers who have only had a short or intermittent relationship with them. It may also be related to how in tune the carer is with the person with dementia’s current identity and interests (as described in the section on carers forcing a particular kind of engagement) and how open or able they are to accept any changes in the person with dementia if they are present.

## Reducing social isolation & opening up the museum

Core processes:

u. Carers can meet others in similar situations and feel less isolated.
v. The museum becomes a safe place for the dyad to return to in the future.
w. The museum is opened up as an enjoyable and interesting place to visit for previously disinterested professional carers.

Although these sessions do not specifically discuss dementia or difficulties, they can still enable carers to feel less isolated through meeting others in a similar situation:

*And it also helped me seeing people with similar problems and, you know, realising that you’re not alone*.

- *Carer interview, site 4*

A positive experience in the museum could lead to it becoming a ‘safe place’ to return to in the future. Some had visited the museum when they were younger, but now came regularly since attending the sessions, and others now visited the museum café outside of sessions as a place they were familiar with and felt comfortable in. However, they could only return when they had the resources to do so.

The sessions may also open up the museum for professional carers in particular. Professional carers who work at a residential home, for example, do not necessarily choose to come to the museum sessions, but attend as part of their job role (whereas family carers may be more likely to attend out of choice). Some professional carers who were disinterested in the museum, but then enjoyed the session, returned either with other residents or outside of their caring role with their own family. One staff member also described examples of care home staff returning outside of a session with other residents, opening up the museum to people with dementia who had not attended the programme themselves, and suggesting it was perceived as a safe and interesting place to go with the people with dementia they support.

## Links between impacts

Some of these impacts of carers on the model are linked. ‘Session function’ and ‘caring responsibility’ are linked as how carers are able to use the session can depend on the level of caring responsibilities they must take, but ‘session function’ also links to the ‘long-term impact’ of sessions, as enjoying together or shared respite is linked to relationship-building and having new things to talk about. ‘Carers controlling access’ to the programme can impact whether the museum is a place the dyad can return to in the future (‘reducing social isolation’), depending on if the space is *carer-friendly* as well as *dementia-friendly*, or if the carer has the capacity (e.g. finances or time) to return. ‘Preventing engagement’ can be linked to ‘comparisons and losses’ where, for example, the carer is trying to get the person with dementia to engage in a manner more consistent with past interests, preventing the person from engaging in the way they want.

## Discussion

This study aimed to examine the impact of including carers in museum programmes for people with dementia and develop a model of carers’ impact which can be used, and developed further, in other kinds of programme. The work refined theories from a previous review (Kinsey et al., 2019), for example the idea of shared respite was expanded to include different kinds of session function. We found the inclusion of carers can have both positive and negative impacts on the person with dementia, the carer, and their relationship. The model suggests these impacts fall into seven broad areas – caring responsibility, session function, controlling access, preventing engagement, comparisons and losses, long-term impact of in-the-moment activities, and reducing social isolation and opening up the museum.

The concept of ‘dementia-friendly’ practices has received increasing attention (Alzheimer’s Disease International, 2017). This research emphasises the need to embed ‘carer-friendly’ practices within dementia-friendly practices, particularly as carers often control access to activities. Currently, there are policies within the UK related to making settings or services ‘dementia-friendly’ or ‘carer-friendly’, but they are treated as separate issues, despite caring being relational. These practices need to be integrated where appropriate, considering that carers and people with dementia will also have specific needs or a desire for their own spaces. This is particularly important where carers are required to attend a programme or activity, as their needs must also be considered, even if the sole focus of the programme is on the person with dementia. For example, one of the reasons there can be a ‘shrinking social world’ (Duggan et al., 2008) in dementia is due to difficulty with unfamiliar places or people, or, from the carer’s perspective, not knowing how the person with dementia will respond and all the caring tasks that go alongside. As the Site 5 manager in this study found, if the programme content is focused on making the unfamiliar comfortable for the person with dementia, without considering how it is unfamiliar to the carer, neither attends and their social world is not expanded by attending the museum.

This study supports previous research suggesting there are many forms of respite, beyond simply carers having a physical break from the person they support (e.g. Chappell et al., 2001), and that respite can be experienced by the person with dementia and professional carers, such as in shared respite (Burnside et al., 2017). For example, family carers can experience ‘role engulfment’, in which the number of caregiving responsibilities leaves little time for other activities and a so there is a loss of personal identities other than ‘carer’ (Eifert et al., 2015). It may be that internal moments of respite in activities, such as the museum sessions, help the carer to re-discover or maintain their other personal identities, while also being ‘carer’. However, for some carers this may link to a realisation of loss and a grief reaction, particularly when they do not have resources to find other ways of maintaining those ‘non-carer’ identities. Further research could usefully consider the relationship between different forms of respite and identity, and the ways in which dyads and individuals could be supported to maintain their identities through other forms of respite. Additionally, given that the low uptake of respite services may be due to carers conceptualising respite differently than policymakers (O′ Shea et al., 2019), policy could expand to include (and fund) different forms of respite. Programmes such as these could form a part of that.

### Practice implications

• The presence of carers has an impact and practitioners should consider how this will affect their programmes. Carers should be viewed as participants of programmes, and can even be the main beneficiaries, even where the programme is ostensibly ‘for’ the person with dementia – it’s not simply that carers are the enablers of, or barriers to, impacts on the person with dementia.
• All programmes in this research gave staff basic training in dementia and potential support needs; training in carers’ needs could be integrated with this. Programme designers should be aware of structural issues related to carers’ needs and of what may make the programme ‘carer-friendly’, such as support with travelling to the session.
• Staff need to be aware people use sessions in different ways, and not all dyads want ‘togetherness’ even where this is the aim. Staff may need to provide more support to carers where they previously enjoyed the session separately, but now must increasingly stay with the person with dementia to support them as their needs increase.
• Staff should be aware carers can dominate group conversations through their enthusiasm and because people with dementia may find it more difficult to speak in a group. Appropriate facilitation strategies can help promote equal participation.
• Programmes may open up the museum as a safe place for the dyad to return to, or for previously uninterested professional carers. Information on how to return and on other activities should be available. Museums could increase people’s capacity to return though schemes such as reduced transport costs.

### Strengths and limitations

Having six sites, including a variety of museum types and locations, and studying existing programmes rather than creating a short-term programme solely for research, strengthens the generalisability and validity of the findings. The resulting model is general enough to be tested and applied in a variety of dyadic programmes.

There were few interviews with people with dementia and carers due to recruitment difficulties. We compensated for this in part through triangulation with session observations and studies from the original literature review, but future research needs to test the model directly with more attendees. The diversity of the sample is limited by the diversity of session attendees, as there were no same-gender dyads who were also couples, and very few non-white dyads. This research cannot be generalised to all dyads, as there may be different or additional processes involved for dyads with different personal characteristics, which may be particularly important in considering what makes a programme ‘carer-friendly’. There was also no long-term follow-up to see if potential long-term impacts were robustly supported.

### Further research

Further research could test the model/theories with more dyads in museum programmes for people with dementia, in other dyadic programmes for people with dementia, and in programmes for people with other support needs. For example, the museum programmes included in this research aimed to promote equal participation for the both the carer and the person with dementia. Investigating whether, and how much, the model applies to programmes which do not have these overarching aims would enable understanding of the extent of its generalisability. The model developed here could then form the basis of robust middle-range theory on the impact of including carers, which would have relevance to a range of programmes and participant types.

Further research could also examine the individual processes in more depth. A large number of programme theories were included in this study, partly due to the lack of previous work in this area. Although this was a strength, it meant some depth about individual processes was sacrificed for breadth. Future research could take just one process, or group of processes, and study those in more depth, rather than applying the whole model. More research is also needed more generally on how the dyadic relationship may impact programme experiences, to better understand how museum programmes could build relationships, including for professional carers.

## Conclusion

Including carers in programmes for people with dementia may at times seem like a convenient fix for issues related to risk or responsibility. Their inclusion may have both unanticipated benefits and negative consequences, and greater attention is needed on how both carers and people with dementia can be supported in shared sessions. Alongside making venues and programmes more dementia-friendly, there must be consideration of how they can become more carer-friendly. Further research could build on the model presented here to create robust middle-range theory on the impact of including carers, which could be used to develop dyadic programme components that would increase the benefits for a range of participants and within different settings and programme types.

## Supplemental Material

Supplemental Material - A conceptual model of the impact of including carers in museum programmes for people with dementia Click here for additional data file. Supplemental Material for A conceptual model of the impact of including carers in museum programmes for people with dementia by Debbie Kinsey, Noreen Orr, Rob Anderson and Iain Lang in Dementia

Supplemental Material - A conceptual model of the impact of including carers in museum programmes for people with dementia Click here for additional data file. Supplemental Material for A conceptual model of the impact of including carers in museum programmes for people with dementia by Debbie Kinsey, Noreen Orr, Rob Anderson and Iain Lang in Dementia

Supplemental Material - A conceptual model of the impact of including carers in museum programmes for people with dementia Click here for additional data file. Supplemental Material for A conceptual model of the impact of including carers in museum programmes for people with dementia by Debbie Kinsey, Noreen Orr, Rob Anderson and Iain Lang in Dementia
