# Supplementary material for: A conceptual model of the impact of including carers in museum programmes for people with dementia
Source: Dementia (London). 2022 Sep 22;21(8):2584–600. doi: 10.1177/14713012221126803 (PMC9583285; doi:10.1177/14713012221126803)
Supplement: Supplemental Material - A conceptual model of the impact of including carers in museum programmes for people with dementia [file sj-pdf-1-dem-10.1177_14713012221126803.pdf]

Supplementary File 1: List of evidence-based programme theories

In a realist approach to evaluation, causal processes are expressed as programme theories in which outcomes are understood to be generated by the interactions between aspects of the context and underlying mechanisms. Mechanisms include the resources offered by the programme, and the participants' reasoning in response. Wherever possible, the mechanism in these theories have been split into its specific resource and reasoning components. The following abbreviations are used throughout: (C) = Context; (M/Rea) = mechanism resource; (M/Res) = mechanism reasoning; (O) = outcome.

**Theory number**	**Refined programme theory**
1	When the carer wants to enjoy the session separately (C), high staffing levels (M/res) mean the staff are able to support the person with dementia when needed (M/rea), so the carer has fewer responsibilities (O) and is able to enjoy the session alone (O).
2	When the carer wants to enjoy the session separately but the person with dementia wants to be close to them (C), then either:<br>a) High staffing levels (M/res) mean the staff support the person with dementia (M/rea), so the carer can enjoy the session alone (O) but the person with dementia is unhappy or stressed (O).<br>b) The carer supports the person with dementia (M) so the dyad stays together (O), but the carer is unhappy or stressed (O).
3	When the carer has always enjoyed the session through being separate (C), but the person with dementia's support needs have increased as their dementia progresses (C), the carer provides more support in the session (M), and is unhappy or stressed as they can no longer use the session for traditional respite (O).
4	In the context of the carer being the one who finds out about the programme and decides whether to attend (C), the person with dementia (dyad) does not attend (O) when the programme does not provide the resources needed to support the carer to feel comfortable or able to attend (M).
5	Where the carer is trying to hold on to the person with dementia's previous identity/interests (C), they try to make the person with dementia engage in a particular way tied to this (M). The person with dementia does not want to (M) and feels annoyed and cannot engage in the way they want to (O).
6	Where the dyad is unsure of new activities to try or often does the same type of activities (C), coming to a new/different kind of activity that the person with dementia enjoys/engages in (M), means the carer seeks out similar activities or replicates them at home (O). In care home groups, the activity is replicated with other residents who did not attend the session (O), extending the impact to those people with dementia outside of the session itself.
	Where the dyad's home interactions are mainly about caring tasks or with limited leisure activities (C), doing a shared activity outside of

7	caring routines (M) means they have new things to talk about and share with others after the session (as well as during) (O).
8	Where carers are unsure if the session is for them or are feeling stressed at the beginning of the session (C), museum staff serving them tea (M/res) helps carers to feel they can also participate, they do not hold all of the responsibility in the session, and gives them space to relax (M/rea). The carer then feels less stressed and welcomed (O).
9	When the facilitation enables the person with dementia to 'maximise capacity' to participate in an activity outside of their usual routine (M/res), the person with dementia responds in a new or unexpected way (M/rea). Where the carer's expectations of the person with dementia are low (C), and/or others in the group highlight the person with dementia's capabilities (C), the carer sees the individual person with dementia in a new way (O) or reconsiders the activities in which people with dementia in general are capable of engaging (O).
10	Professional carers who only have a relationship with the person with dementia in a work context with limited opportunities for shared or social activities (C), take part in a shared activity outside of the work context (M/res) and are able to get to know the person with dementia beyond dementia (M/rea), which builds their relationship.
11	Where dyads do not know each other well, or the person with dementia has changed some of their interests or aspects of their identity (C), sharing an experience outside of their usual routine on an equal basis (M/res) means they have meaningful or new communications and interactions (M/rea) which helps the carer to see the person with dementia in a new way (O) and build their relationship (O).
12	In dyads where the carer does not know the person with dementia well (C), is unsure how to support the person with dementia (C), or has fallen into habitual patterns of interacting (C), staff interacting with the person with dementia in a new way (M/res) means carers learn new ways to interact with them and new strategies (M/rea), which builds their relationship through improved communication and a focus on process rather than product (O).
13	Where the person with dementia has anxiety, or is anxious about new activities or new people (C), the carer reassuring or modelling taking part in a shared activity (M/res) helps the person with dementia to feel comfortable/able to take part (M/rea), so the person with dementia is able to participate without anxiety (O), but the carer cannot be separate within the session (O).
14	Where the dyad's home interactions are mainly around caring and they want to spend time together (C), enabling facilitation of an activity not about dementia in a community setting, in which the carer only has to provide the level of care for the person with dementia that they want to (M/res), means the dyad can participate on an equal basis (M/rea), which leads to the dyad experiencing shared respite from dementia/caring roles (O), which also helps strengthen their relationship (O).
	Shared respite (C) means the dyad can interact and communicate outside of dementia roles (M), which builds their relationship (O) and also

15	feeds back into enjoying the activity together (O).
16	The participatory activity with a ‘product’ that is outside of their daily routine (M/res), in the context of high carer expectations or a dyad struggling to accept dementia (C), means the family carer compares the person with dementia’s current abilities to their former abilities or with other people with dementia in the group (M/rea), which leads to the group highlighting losses or reinforcing limitations (O).
17	Where the family carer feels respite in the session (C), they realise they are missing other activities they enjoy (M), and this highlights personal losses for the carer (O).
18	When personal losses are highlighted for the family carer (C), they realise they need to find activities just for them and seek them out (M), and then join other groups without the person with dementia (O) if they have the capacity to do so.
19	Where the family carer has high expectations of the person with dementia (C) or the group highlights losses (C), the carer does not perceive the person with dementia’s capabilities or feels upset (M), so the carer does not feel respite (O) and does not see the person with dementia in a new way (O).
20	When the carer has high levels of caring responsibilities in the session (C), they cannot engage with the activity (M), so they do not feel respite or enjoy the activities (O).
21	Anxious carers (C) intervene on the person with dementia’s behalf (M), so the person with dementia cannot engage fully (O) and the carer has no respite (O).
22	Where the person with dementia struggles with speaking or finds it stressful (C), the carer answers for them in the group or translates their answers for others (M), so the person with dementia is able to engage in a way which is comfortable for them (O).
23	Professional carer-person with dementia dyads have only had a caring/work relationship so find it harder to step out of caring roles (C), or museum staff perceive professional carers to only have a caring, and less participatory, role (C). This means the carer intervenes on the person with dementia’s behalf or does not participate themselves (M), and the person with dementia and/or professional carer cannot engage fully (O) or gain potential relationship-building outcomes (O).
24	Where there is poor facilitation or the carers are particularly enthusiastic about the activity (M/res), and the person with dementia finds it more difficult to speak up in a group setting (C), carers dominate group conversations (M/res), so the person with dementia cannot engage verbally, does not have a sense of belonging in the group, and cannot gain potential positive outcomes (O).

25	Where carers are not in contact, or have limited contact, with other carers (C), the activity happening in a group means carers meet others in similar situations (M/res) and feel connected to others in a similar situation and the wider community (M/rea), so feel less socially isolated (O), and leads some to continue friendships outside the group (O) or join other dementia-friendly groups (O).
26	A positive experience in the museum (M/res), in the context of a 'shrinking world' for the dyad (C), makes the dyad feel the museum is a 'safe place' they can return to (M/rea), so they intend to return outside of the group (O) and feel less socially isolated (O).
27	Professional carers who would not choose to come to the museum and are not interested in it (C), experience an engaging, participatory session where carers are included (M/res), so feel the museum is an enjoyable or interesting place (M/rea), and return outside of the session (O). They return in two ways:<br>a) Personally outside of work roles (such as with family)<br>b) Professionally with other residents who did not attend the session (the museum becomes perceived as a good place to visit with the people with dementia they support more generally, and the sessions indirectly open up the museum to people with dementia who did not attend a session themselves).
28	A positive experience in the museum (M/Res), in the context of a 'shrinking world' but where the carer does not have capacity to include leisure in day-to-day life or who need the additional logistical support of a session (C), means the carer does not see the museum as a place they can return to outside of sessions (M/rea), so they do not intend to return and do not feel less isolated (O).
