# Supplementary material for: A conceptual model of the impact of including carers in museum programmes for people with dementia
Source: Dementia (London). 2022 Sep 22;21(8):2584–600. doi: 10.1177/14713012221126803 (PMC9583285; doi:10.1177/14713012221126803)
Supplement: Supplemental Material - A conceptual model of the impact of including carers in museum programmes for people with dementia [file sj-pdf-2-dem-10.1177_14713012221126803.pdf]

Supplementary File 2. Revisions of the review theories with evidence

This document gives detailed information on evidence for each of the developed programme theories. Theories 1 to 8 are new theories developed from the evaluation, and theories 9 to 28 are theories revised or developed from the original literature review (Kinsey et al., 2019). Where theories were revised or developed from the review, the original theory is first presented, followed by evidence from the evaluation, and then the final revised or newly developed evaluation theory.

Contents

Theories 1 -3 1

Theory 4 7

Theory 5 8

Theories 6 & 7 10

Theory 8 11

Theory 9 – (review theory a) 12

Theory 10 – (review theory b) 15

Theory 11 – (review theory c) 16

Theory 12 – (review theory d) 18

Theory 13 – (review theory e) 20

Theory 14 – (review theory f) 21

Theory 15 – (review theory g) 24

Theories 16-18 – (review theory h) 25

Theory 19 – (review theory i) 28

Theory 20 – (review theory j) 29

Theory 21 & 22 – (review theory k) 30

Theory 23 – (review theory l) 32

Theory 24 – (review theory m) 34

Theory 25 – (review theory n) 37

Theories 26 & 27 – (review theory o) 39

Theory 28 – (review theory p) 41

References 43

Theories 1 -3

As described in the paper introduction, one of the common aims of these museum programmes is an ‘enjoying together’ for the dyad, and one of the key positive outcomes outlined in the review was the idea of shared respite – a break, together, away from dementia and their roles as carer and cared-for. However, from both observations and interviews, it is apparent that a person with dementia and their carer can use the session in different ways. Whilst some enjoy the session

together, for others the aim is not this ‘enjoying together’ but enjoying *separately*, much like a more traditional model of respite.

During my analysis, I coded how dyads were participating in sessions, whether together or separately. I coded ‘enjoying together’ as distinct from the dyad having to be together due to the person with dementia’s needs and/or due to caring responsibility.

There was evidence of dyads enjoying the session together in fieldnotes from all six museum sites and from descriptions in six interviews (one with a professional carer-person with dementia dyad at site 4, and five with staff at sites 1, 2, 4, and 6). For example:

> *Another woman who used to come with her mother, and they used to pretty much stay together and say that they really enjoyed being able to do something creative that was in a supportive group but that they could do together.*
> *- Manager-facilitator interview, site 2*

> *…Fiona [facilitator] talked a bit about that, and the Australian links between the painting and the BBQ. Pearl [person with dementia] said “Australian” in an Australian accent to Naomi [carer] and they both laughed. Fiona talked to Naomi and Pearl about the accent, while Stella [person with dementia] and Robert [carer] talk to each other about the BBQ, also making jokes and laughing.*
> *- Fieldnotes, site 1*

There was also evidence of dyads enjoying the session separately in fieldnotes from all sites except site 5, and in descriptions in five staff interviews at sites 1, 2, 4, and 6.

> *When we walked over to [building], a member of museum staff pushed Beryl’s [person with dementia] wheelchair. Throughout the session Beryl and Louisa [carer] were separate, with the member of staff talking to Beryl and involving her in the session…*
> *– Fieldnotes, site 4b*

> *During tea, [the dyad] sat at the same part of the table, but each on one side of the table’s corner and mostly talked to other people in the group or staff. During the session they very rarely walked together between objects and never sat together at objects. They talked to other people in the group but rarely to each other.*
> *– Fieldnotes, site 6*

Having high staffing levels, relative to other kinds of museum programmes, appeared to support dyads to enjoy the session separately. In fieldnotes at all sites except site 5, and in interviews with seven members of staff from sites 1, 2, 4, and 6, having high staffing levels appeared to be a resource which allowed staff to support individual people with dementia (and carers) when needed, taking some of the responsibility of support from the carer and enabling them to be separate in the session.

> *…because we’ve developed quite high staffing levels because we realised that that was important and also necessary, it has often been the case, I think, that particularly family member carers have been able to actually have a bit of space in the group. And that the person that they’ve come with has been, you know, we’ve been able to support them and chat to them and, you know, give them quite a bit more time. And so if it’s a partner, for example, they’ve been able to be a bit more independent and have a chat to the curator or wander around a different part of the gallery and know we are there to support their partner.*

– Manager-facilitator interview, site 2

Volunteers helped [person with dementia] to stand as she was struggling with the slippery floor. [Volunteer] moved her chair and helped her to sit back down again. [Carer, her husband] was talking to [other carer] on the other side of the room.
– Fieldnotes, site 1

However, staff also discussed limits on what responsibility they could take, for example they could not support the person with dementia to go to the bathroom. This meant staff could support the person with dementia, enabling the carer to enjoy the session separately if they wished, until the person with dementia's needs reached a certain level or type, or the person with dementia wanted the carer specifically to support them.

I think for a start we realised involving carers is important because it removes the barrier of like…the safeguarding barrier from us. Because otherwise our [facilitators] would have to…they would have to have a lot of…a lot more training, and we don't want to put that much pressure on any of our staff, that they are carers.
– Manager interview, site 5

I don't think I'm meant to be able to push wheelchairs for health and safety reasons.
– Facilitator interview, site 4

At times there was a mismatch in the way the person with dementia and carer wanted to use the session. For example, at site 1, there were two clear instances where there seemed to be this mismatch in the desired session function. In the first, the carer could not be separate as the person with dementia wanted their support. In the session, Robert [carer, husband] did not stay with or assist Stella [person with dementia, wife] unless she specifically asked for his help, and he always left her again as soon as he had helped her, whereas she would walk to stay close to him. For example:

Stella was struggling to get out of the chair. I held the back of it but that didn't help enough. She looked over at Robert and said 'I can't get out of here'. Robert came over and helped her stand up. As soon she was standing, he told her he was going to look at the photographs and walked to the other side of the gallery. Stella stood with us while we folded chairs, and then walked over to Robert.
– Fieldnotes, site 1

In the second, the carer was separate in the session with the person with dementia supported by staff, but the person with dementia was stressed and unhappy that they were not with the carer. This was also observed at site 2:

[Facilitator] said that during the drawing activity James [person with dementia] seemed to struggle and after starting to make some marks he sat and didn't participate. [Facilitator 2] said she had noticed Jo [carer, wife] and James didn't walk around the gallery together, and didn't sit together during the art activity. She said she thought James wanted to sit with Jo but Jo didn't want to.
– Fieldnotes, site 2

As stated in the analysis section, I tracked dyads who appeared in more than one piece of data (such as more than one set of fieldnotes or fieldnotes and interview transcripts) to see if the function of sessions fluctuated for individual dyads over time. Nine dyads across five sites could be included in this analysis, including two professional carer-person with dementia dyads as described by staff in meetings and interviews rather than through observations. They are summarised in Table 1 below.

Table 1. Summary of dyad tracking

Site	Dyad type	Appearances in the data	Brief notes on session function
1	Family<br>(mother & daughter – daughter as carer)	Attended 1 of 5 sessions and mentioned in 1 staff interview	- Always together as person with dementia needs physical support and reassurance.<br>- Enjoy together and a number of shared moments.<br>- Person with dementia does not seem consistently engaged in the session. Carer often talks to facilitator and others in group when person with dementia is not engaged.
	Family<br>(spouses – wife as carer)	Attended 2 of 5 sessions	- Always together as person with dementia has high physical needs and person with dementia only responds to his wife.<br>- Person with dementia does not seem very engaged, but carer enjoys talking to others in the group and staff.<br>- Despite high levels of caring responsibility, sessions seem to be more for the carer to enjoy.
	Family<br>(spouses – husband as carer)	Attended 4 of 5 sessions and mentioned in 2 staff interviews	- Always enter separately. Carer takes every opportunity possible to be separate from her (and at times the whole group), only supporting the person with dementia when staff are struggling or she specifically asks for his help. After helping her he leaves to go elsewhere in the gallery or expresses frustration.<br>- Do enjoy together at times through joking and teasing (initiated by both)<br>- Person with dementia is engaged in the sessions but less so than the carer.<br>- He wants to keep attending after she dies, so it is not just a place he can have respite, but something he genuinely enjoys for himself.<br>- Described as being one of increasingly fewer places he can persuade his wife to go.
2	Family<br>(spouses – wife as carer)	Attended 3 of 4 sessions. Mentioned in 3 meeting notes and 1 staff interview.	- Always separate in sessions, though at least once the person with dementia wanted closeness where the carer did not.<br>- Staff give person with dementia support to do the activities, so carer can do it separately.
	Professional<br>(care home)	Attended 2 of 4 sessions (different professional carer each session). Mentioned in 1 meeting note and 1 staff interview.	- Person with dementia is a wheelchair user so have to be together as museum staff not allowed to push wheelchairs.<br>- Enjoying together in sessions – talking about their reactions to the activities.
	Professional<br>(care home) and family (siblings –	Attended 3 of 4 sessions (professional	- Person with dementia initially spent time alone in sessions (including away from museum staff), but in the final session stayed more with the group and

	sister as carer)	carer for first two and sister in the third). Mentioned in 2 meeting notes.	spoke more to others.<br>- Professional carers report he likes to be independent at home as well.<br>- Person with dementia does not need much support in sessions so able to choose to be separate or close.
3	Family (mother & 2 daughters – daughters as carers)	Attended 1 session and mentioned in 1 staff interview.	- Always together as person with dementia needs physical support.<br>- At a previous museum they were more separate as person with dementia did not need as much support.
4	Family (spouses – wife as carer)	Attended 2 sessions (1 set of fieldnotes) and each did an individual interview.	- Always together in sessions (not due to person with dementia's support needs)<br>- Carer much more interested in the sessions, but person with dementia 'happy to go along'<br>- Less of a 'shared experience' as he is not as interested, but was a way of getting out together when both hesitant to do so after initial diagnosis.
6	Family (spouses – wife as carer)	Attended 3 sessions (2 sets of fieldnotes)	- Always enjoy the session separately. Both enjoy it and talk to others but rarely with or to each other. Carer often irritated when they do speak.<br>- Staff stay with the person with dementia when he wants to take a different or slower route through the museum than the rest of the group. He has no physical or mental support needs in the session beyond this.

All dyads were generally consistent in the way they used sessions and session function did not appear to fluctuate. Two staff members (at sites 2 and 4) also described session function as being consistent. However, this may be due to the relatively short period of time spent at each programme, as there were indications that session function could change as the person with dementia's dementia progresses or they need greater support from their carer. For example, at site 1, one dyad (Robert, carer, and Stella, person with dementia, spouses) appeared to enjoy the session separately, with Robert in particular using it to have time to himself to enjoy the museum. During the research, Stella's support needs, both in terms of physical support, such as sitting and standing, and mental support, such as reassurance, gradually increased. Robert increasingly needed to support Stella, particularly as she wanted the support from him rather than from staff. He often expressed frustration and would immediately move away to be on his own after supporting Stella.

This transition period, when a carer who uses the session for more traditional respite but then must increasingly be with the person with dementia to support them, may be challenging for some carers. The function of the sessions for the carer has been forced to change due to caring responsibilities at a time when their caring responsibilities outside of sessions may also have increased (and so at a time when they may feel even greater need for the more traditional respite for which they usually come to the sessions).

This interaction of caring responsibilities with the function of sessions for the dyad as a whole, or for them as individuals, is complex. For example, some carers enjoyed the session in a 'shared respite' way while needing to be with the person with dementia due to their support needs (i.e. there is no choice for the carer in being together). In the following example, Louisa (professional carer) had to

stay with Jane (person with dementia) to push her wheelchair and support her to do the activities, but they enjoyed the session together:

> *[Facilitator 3] said Louisa and Jane seemed to be really enjoying it together, like they were a team. Jane had a magnifying glass and they were chatting the whole time about different things sparked off from the illustrations. And during the drawing Louisa helped Jane by repeating the instructions for her, and they both talked about their drawings and laughed together afterwards. [Support staff member] said Jane was very enthusiastic when they left, and Louisa said she was 'glad it was something they did together'*
> *– Fieldnotes, site 2*

Whereas, for others, the carer only seemed to enjoy the session if they were able to have no responsibilities. In this session, the museum staff supported the person with dementia to do the activity while his wife, his carer, sat on the other side of the table and completed her own artwork:

> *[Facilitator 2] said that [person with dementia, husband] and [carer, wife] do a lot of activities together, but she always has to be looking out for him, so she can't always enjoy it for herself. At the museum she knows we will look out for him so she can relax herself too.*
> *– Fieldnotes, site 2*

At other times, the carer enjoys the session as a more traditional respite, with it seemingly more for the carer than for the person with dementia, despite the fact they must be with the person with dementia at all times to support them. For example, one of the spousal dyads included in the site 1 dyad tracking was George (person with dementia) and Lucy (his wife). Despite Lucy having to be with George at all times due to his support needs, the sessions appeared to be more for her to enjoy as George was usually not engaged with them. They seemed to be a form of respite through the activities themselves and through having conversations with others that she could not have with George. This fits with the idea of 'internal respite' in Chappell, Reid, and Dow's (2001) research examining carers' (including, but not exclusively, those caring for people with dementia) conceptualisations of respite. 'Internal respite', according to this study, is where the carer feels they are taking a break whilst remaining in the caregiving situation, and can include 'stolen moments' (something that is not an absolute break from caring but is a routine task such as taking a shower, or caregiving in a new enjoyable setting) and connections with others. In the case of Lucy and George, for example, Lucy may have experienced an internal respite through connection and stolen moments (specifically being a new setting and activity). However, evidence for this was limited to session observations and connections to the literature, as I was unable to interview any carers who seemed to be experiencing the session in this way.

What the interaction between levels of caregiving, session function, and outcomes means for developed theories will be discussed further in examination of the results as related to theories 14 and 20.

This section discussed findings on the function of sessions for dyads, particularly that some want to use the session as a more traditional form of respite (enjoying separately) rather than as a shared respite, and that there can be mismatch in how the two people in the dyad want to use the session. From these findings, three new theories were developed related to session function:

1. When the carer wants to enjoy the session separately (C), high staffing levels (M/res) mean the staff are able to support the person with dementia when needed (M/rea), so the carer has fewer responsibilities (O) and is able to enjoy the session alone (O).

2. When the carer wants to enjoy the session separately but the person with dementia wants to be close to them (C), then either:

    a. High staffing levels (M/res) mean the staff support the person with dementia (M/rea), so the carer can enjoy the session alone (O) but the person with dementia is unhappy or stressed (O).

    b. The carer supports the person with dementia (M) so the dyad stays together (O), but the carer is unhappy or stressed (O).

3. When the carer has always enjoyed the session through being separate (C), but the person with dementia's support needs have increased as their dementia progresses (C), the carer provides more support in the session (M), and is unhappy or stressed as they can no longer use the session for traditional respite (O).

Theory 4

Related to the ways carers supported the person with dementia in the session if they needed it, carers also enabled the person with dementia to attend the session through:

a. finding out about the session and booking

Both people with dementia who were interviewed stated it was the carer who found out about the sessions, including one person with dementia who lived across the road from the site but it was her daughter who lived much further away who found them.

b. dealing with the logistics of getting to the session

This included managing travel, parking, and navigation. One programme manager discussed how it could be harder for family carers to attend for this reason, as care homes often have a member of staff whose role is to manage these logistics while family carers do not have this support.

> *Person with dementia: Because a lot of us, obviously we have people, like I've got [carer], I've got my family, and some people haven't got anything, so they'll never get to [museum] because it's not advertised or anything. There's not enough space.*
>
> *Carer: Yeah you wouldn't have found out about it without [daughter] finding out about it, would you? You wouldn't have known where to look.*
>
> *Person with dementia: No. Absolutely not.*
>
> *– Professional carer & person with dementia joint interview, site 4*

As the carer is the one who found out about the session and booked, they effectively controlled whether the dyad (and so the person with dementia) attended. There can be a 'shrinking social world' in dementia is due to difficulty with unfamiliar places or people, and, from the carer's perspective, not knowing how the person with dementia will respond and all the caring tasks that go with that (Duggan et al., 2008).

> *But [facilitator] said it's a real shame, having now delivered something and knowing that there are things out there for people to experience in safe environments, in welcoming environments, that her friend's [carer for husband with dementia] reaction was that the only way she could cope with it was just to keep him at home all the time.*
> *– Manager interview, site 3*

The manager at site 5 talked at length in her interview about how much research and planning went into making the sessions dementia-friendly, including talking to a number of carers about how to adapt activities for people with dementia's needs. During the period of the research, these sessions were very poorly attended, with usually only a few participants or cancelled due to lack of bookings. At DK's final session at site 5, the manager said she had recently delivered an outreach session to carers (mainly professional but with some family carers) in which she discussed the tours, what happened on them, and demonstrated example activities. She said the carers told her they would not have brought people to the tours prior to the outreach session as they had never been to site 5 and were not sure how suitable the tours or setting would be. The bookings for tours following the outreach session did then increase. This suggests programmes also need to focus on what makes it accessible or comfortable for carers, not just people with dementia, even where the person with dementia is the main focus of the session and the session is primarily for the person with dementia.

Two programmes (at site 4 and 5) sent out information to carers following booking on what to expect in the session and the logistics of getting to the museum. Site 4 also included a pre-session telephone call with the manager to give further information, answer any questions, and for the manager to find out if the carer or person with dementia had any particular support needs. The managers at site 4 and 5 both felt this was an important way of supporting carers and helping them to feel comfortable attending. However, no museum site provided this level of information in advance of booking, so it would not help with those carers who felt too unfamiliar or unsure of what would happen to book in the first place. This is exemplified by the carers in the outreach session at site 5 saying they would not book without that information, only offered by site 5 after booking. It may suggest that publicity or marketing materials need to be tailored to what the carer needs to know to feel comfortable attending, particularly if they have not visited the site previously.

This evidence around carers controlling attendance led to the development of a new theory:

4. In the context of the carer being the one who finds out about the programme and decides whether to attend (C), the person with dementia (dyad) does not attend (O) when the programme does not provide the resources needed to support the carer to feel comfortable or able to attend (M).

Theory 5

One part of the ethos of these programmes, is the idea of the participant's identity and meeting them 'in the moment' – not who they were in the past or what they were interested in then, but with interaction based on who they are and what they are interested in on the day of the museum visit. This is enacted in the sessions through the way activities are structured and facilitated, such as not focussing on reminiscence or memories but on the person's response to a particular piece of artwork, for example.

However, there was evidence of some carers struggling with this. There were occasions, both observed in sessions and from descriptions in staff interviews, where carers tried to prompt a particular memory or shared memory linked to the activity or discussion from the person with dementia. There was never a negative response from the person with dementia when this happened during sessions I attended. When it seemed like the person with dementia could not remember what the carer was trying to prompt, they usually just answered with a simple yes or no, changed the subject, or walked away.

[Carer] turned to [person with dementia] and said 'look! [Place name]! You used to live there.' [Person with dementia] looked unsure and said 'oh yes' and continued walking…[Carer] turned back to me and started talking about [place name].
– Fieldnotes, site 3

…sometimes carers have expectations, they'll say, 'oh my husband used to be a painter, he really wants to see paintings' or 'my husband used to be a carpenter, we have to find him some wood'. And that actually what you find is that often when every time we've tried to do that with people, so…we did have those specific examples one time….And the man who was a carpenter, one of the curators let him touch some Victorian wood carving. He didn't actually recognise that it was wood. But there were some modern ceramics that he really loved looking at and touching. So I feel like the process of seeing something that you like that day may or may not be anything to do with your previous identity.
– Manager interview, site 1

At times the carer tried to make the person with dementia engage in a particular way with the activity or discussion, based on things they used to enjoy or an aspect of their identity. For example, in one session at site 4, a person with dementia who used to be a musician did not want to get actively involved in a music-making activity and said he just wanted to listen. His wife (his carer) had mentioned earlier that they had sold his instruments as he was no longer able to play them – so the person with dementia may not have wanted to get involved due to a grief for that skill or because he was no longer interested. However, the carer persisted in trying to get him to make music, and later to answer music-related questions. The person with dementia became increasingly annoyed with his wife during the session.

In dyad 1, the husband [person with dementia] said something quietly to himself, and his wife [carer] nudged him and loudly told him to speak up to the group. He shook his head and said no, that he didn't want to. His wife kept telling him to speak up. The husband seemed irritated and said he didn't want to and that he wanted to be left alone. During this time the group conversation had identified the composer and piece of music. The wife said to the group 'we've got [incorrect composer name] over here', and the facilitator said 'that's very close, it's [name]'. The wife turned to her husband and said 'oh it was [name]' and at the same time the husband said in the same irritated tone as before 'I didn't want to'.
– Fieldnotes, site 4a

It may be that, in some cases, the way the mechanism of 'in the moment' ethos on identity, and the way the staff enact that, does not have enough leverage to overcome the context of the carer (Jagosh, 2017). The carer may have had a long, close relationship with the person and so has a very strong idea of who they are compared to much more rapid changes related to dementia. This may be combined with a grief for the way their lives were, or who the person was, before dementia (Blandin & Pepin, 2017) which is not easily overcome in a two-hour museum session.

A new theory was developed on this potential negative impact of including carers:

5. Where the carer is trying to hold on to the person with dementia's previous identity/interests (C), they try to make the person with dementia engage in a particular way tied to this (M). The person with dementia does not want to (M) and feels annoyed and cannot engage in the way they want to (O).

Theories 6 & 7

This evaluation did not include a long-term follow-up of dyads outside of the sessions to assess any long-term impacts. However, there were two potential long-term impacts of including carers suggested by the data.

a. Seeking out or replicating the activity

Staff from care home groups at site 2 were described as intending to replicate the art-making activity in the care home with other residents:

> *There's quite a lot of examples of [professional] carers going 'oh we never thought of approaching something like that, that's really interesting to do that kind of drawing activity' or 'we didn't know people would receive it like that and you know we could go back and do some of these kinds of things'*
> *– Manager-facilitator interview, site 2*

And family carers were described by a manager at site 4 as seeking out other similar activities as the person with dementia had enjoyed their experience in the museum:

> *And sometimes we'll do an activity and perhaps a carer will say something like 'oh my partner really enjoyed doing X, that's great for me to know because I'll try and find more things that we can either do at home that's doing that, or going elsewhere to do that'. It might be like painting, or listening to a certain type of music, or they might have used a musical app on the iPad and think 'oh well I'll try and download that because my partner really enjoyed making music that way'. So that's always nice to see.*
> *– Manager interview, site 4*

By including carers, who, as previously discussed in Theory 4, were the ones who found out and booked activities for the dyad, the person with dementia (and the carer) could then get involved in similar activities elsewhere, or care home residents who did not attend the session could enjoy a similar activity in the home. However, it can only be suggested as an intention of carers, as there was no long-term follow-up to determine if they actually did re-create the activities afterwards.

b. New conversations outside the session

One review theory (Theory 11) suggested coming to the session means the dyad has new conversations, which helps them to learn new things about each other and build their relationship. All but one study included in the development of Theory 11 focused on new conversations within the session. One study (Lamar, 2015) asked carers if they had talked with the person with dementia about their museum visit afterwards, finding that some did but others could not due to the person with dementia's lack of memory for the session. As this potential long-term impact was not discussed in any depth in the review, it will be discussed here.

Staff interviews and a professional carer-person with dementia dyad in this evaluation suggested attending the session together also gave them new things to discuss outside of the session and new things to share with others.

> *…so to have something that you've had time out as well, that you bring into the relationship, the conversation over support, has to be beneficial I think.*
> *– Facilitator 3 interview, site 6*

> *…instead of having to talk about when people's next hospital appointments are, this issue, that issue, they're able to say 'oh we had a lovely time at the museum and we went to do this shared experience'.*
> *– Manager interview, site 3*

If the person with dementia attended the session alone, they may have had new things to discuss with the carer when they returned home. However, the professional carer-person with dementia dyad at site 4 suggested it helped that it was a shared experience, as they could prompt each other about different aspects of the experience. It may also link with the idea of shared respite from caring roles, as it is a source of conversation unrelated to caring tasks (with the caveat of the varied level of caring responsibilities different carers feel or wish to take within the session).

> *Person with dementia: … I actually very much enjoy listening to it, but if you ask me questions afterwards then I wouldn't be able to deal with that. You can understand that.*
>
> *Carer: But then you always remember if I remind you of things, you do normally remember us doing it in a session.*

And later in the interview:

> *Carer: Yeah we do [chat about it afterwards]. And we often come away with something, and you've got them all laid out in your house, haven't you?*
>
> *– Professional carer & person with dementia interview, site 4*

This therefore led to the development of two new theories about the longer-term impact of including carers:

6. *Where the dyad is unsure of new activities to try or often does the same type of activities (C),* coming to a new/different kind of activity that the person with dementia enjoys/engages in (M), means the carer seeks out similar activities or replicates them at home (O). In care home groups, the activity is replicated with other residents who did not attend the session (O), extending the impact to those people with dementia outside of the session itself.

7. *Where the dyad's home interactions are mainly about caring tasks or with limited leisure activities (C),* doing a shared activity outside of caring routines (M) means they have new things to talk about and share with others after the session (as well as during) (O).

Whether, and how, the second of these theories is linked with Theory 11 will be discussed in evidence for that theory.

Theory 8

All sites except site 3 included a tea and coffee break in their sessions. All but site 5 had their tea at the very beginning of the session, whereas site 5 had theirs following the tour during the object-handling. Although site 3 did not include a tea break, the manager said (unprompted) that it was something she wanted to include, and had done so at a different museum, but could not due to restrictions on food and drinks in the space she was using.

Having tea, particularly tea at the start of the session, appeared to serve a number of functions related to the inclusion of carers. The tea being made and served by staff was one way staff modelled that the sessions were also for the carer and that they did not hold all of the responsibility within the session. It also seemed to set the tone in other ways, by indicating the session was

relaxed and informal. It was also used to welcome the participants to the museum and allow unstructured time for staff and participants to socialise and get to know one another.

> *[Tea and biscuits] sets the tone, you know, that this is going to be fun or it's relaxed and you're welcome here.*
> *– Carer interview, site 4*

> *At [previous museum] the really good thing was there was a hot drink to start with, there was a hot drink at the end, and it really set the tone of this nice friendly place, we're just going to take our time, we're just going to enjoy ourselves. Whereas here, it's a different set-up….I think for the carers that would help as well because they would immediately feel more at ease, whereas at the moment it's kind like 'we're going to begin in the [gallery name] and then we're going to walk down together' instead of getting to know each other a bit I guess.*
> *– Manager interview, site 3*

It was also a way of supporting carers. As described in Theory 4, carers usually had responsibility for getting the dyad to the session, and that these logistical issues could be quite stressful. Having tea at the start allowed space to de-stress after the journey and allowed some gathering time for people who may be late.

> *I think [having tea at the beginning] does a lot of things. It kind of gives, I mean, you never know what journey people have had to get here, that could be really stressful, so just giving them a space where they can just sit down, have a breather, have a cup of tea, go to the loo if they need to, and just kind of collect themselves before going off on a tour I think is quite important. Also being kind of welcomed into the space, familiarising themselves with the [facilitators]. The [facilitators] tend to go round and introduce themselves individually before they gather everyone together….And I think it somehow brings them together as a group before they go off. And hopefully it makes people feel a bit calmer. Because also just finding your way around the building itself is really confusing, so it just builds in that buffer so if people are late we're still here and it's fine and they haven't come all the way to the museum and then are stuck or lost or anything like that.*
> *– Manager interview, site 6*

A theory on serving tea has been developed below:

8. Where carers are unsure if the session is for them or are feeling stressed at the beginning of the session (C), museum staff serving them tea (M/res) helps carers to feel they can also participate, they do not hold all of the responsibility in the session, and gives them space to relax (M/rea). The carer then feels less stressed and welcomed (O).

Theory 9 – (review theory a)

Original theory from the review:

> "When the facilitation enables the person with dementia to 'maximise capacity' to participate (M/res) and *the carer's expectations of the person with dementia are low* (C) and/or others in the group highlight the person with dementia's capabilities (C), the carer perceives the person with dementia to have achieved competency (M/rea), so the carer sees the person with dementia in a new way (O), which also helps build their relationship (O)."

There was some evidence of a shift in carers' expectations of what the person with dementia would be able to do or how they would engage with the activities during the session. For example, in interviews from sites 2 and 3, staff members described occasions of professional carers expecting

less engagement from the person with dementia and being surprised by how much they then did engage. This surprise may suggest the carer's expectations were initially low, supporting the hypothesised context for this theory.

> *…and her carers from the retirement village said "oh you won't get a lot out of her". And at the end she was looking through this magazine and she suddenly said something like "I used to be a…" something like a conglomerate girl or something, I don't know what the word was…it was to do with copying magazines and typefaces, and she said "this would have been very expensive" to [museum support staff]. …. But the fact that they said she won't engage and she did.*
> *- Manager-facilitator interview, site 3*

A manager at site 1 described an occasion where others in the group highlighted the person with dementia's capabilities and that this led to the carer seeing the person with dementia in a new way.

> *So we did have…it was a husband and wife where the husband had been an artist as well. And she didn't let him paint at home anymore because she found the mess too stressful. … And sometimes she would snap at him. And he had dietary requirements and he would try and eat biscuits and she would snatch them away from him. … …we did collage which was not at all the kind of artwork that he used to do. … And we had paper and scissors and he was really confident, he was making these big bright pictures that were…it was like watching Matisse. … …you could see his wife, watching him being praised by other people – genuinely, sincerely – both people with dementia and museum staff, that I think it made her feel differently about him. And also in herself in seeing…like it's great to feel proud of somebody you're connected to.*
> *- Manager interview, site 1*

However, no carers were asked about these aspects of context, so evidence for the context of this theory is still limited.

There was a perception from two members of staff that an enabling facilitation leads the person with dementia to respond in a new or different way, and then the carer increases their expectations of them or sees them in a new way.

> *I think also there are quite long silences often which [carers] feel awkward about, and sometimes it's best just to wait and then the client will come out with something extraordinary. Well, not necessarily, but something you weren't expecting, and that's just a really good way to go. But yeah we have had occasions when you do have to just step in and just "oh just hold on a sec, let's just see what she's going to say".*
> *- Support staff interview, site 2*

The 'maximising capacity to participate' of the original theory may be more specifically this 'responding in a new or different way'. This is also linked with this being a new or different activity to their usual routine, which may also lead to new or unexpected responses from the person with dementia.

> *I can imagine that would be very true that you'd see your partner do things or…and you hadn't known because you'd never been in that situation before and didn't know they enjoyed it or that they could do it or that they wanted to do it.*
> *- Manager interview, site 4*

In both observations (fieldnotes) and interviews, 'enabling facilitation' appeared to be a supportive kind of facilitation – from being responsive and patient with the ways different people tried to communicate, to not making negative comments about participants' contributions, to finding ways to help keep the session focused while giving people the space they need:

> *There's quite a few people there who have a few little quirks about what they say or how they say it, and the people who lead the discussions are just really good at keeping it in focus and not making negative comments, but at the same time moving things on.*
> *- Carer interview, site 4*

> *Pearl [person with dementia] described the shape of the wings using her hands and fingers and said "wings". It was too quiet for [others in the group] to hear, but Naomi [carer] and [facilitator] responded that they liked the wings, and that they seemed difficult to make. Pearl smiled and nodded and repeated the gesture and said "wings" again.*
> *- Fieldnotes, site 1*

One staff interview at site 2 suggested a new outcome related to this theory, and to the idea of replicating activities in the care home with other residents after the session (discussed in relation to Theory 6). She suggested that it is not about professional carers shifting their thinking in terms of what the *individual* person with dementia is capable of, but it is more that their thinking shifts about what is possible in terms of activities with people with dementia more generally, for example the type and complexity of activities people can engage in and enjoy.

> *…also [learning happens] around approaches to activities, you know, there's quite a lot of examples of carers going "oh we never thought of approaching something like that, that's really interesting to do that kind of drawing activity" or "we didn't know people would receive it like that and you know we could go back and do some of these kinds of things". I don't know whether I could confidently say that it alters people's way of communicating…I'd be quite confident to say that there was a shift in terms of people thinking what's possible in terms of the types of activities and kind of creative responses we work with.*
> *- Manager-facilitator interview, site 2*

The mechanism of this review theory includes the carer perceiving the person with dementia to have "achieved competency" as this was the language used in the paper the evidence was drawn from. However, "competency" is a loaded, judgemental term, and falls into negative stereotypes of 'incompetency' which have a negative impact on the lived experience of people with dementia (Scholl & Sabat, 2008). The mechanism might be more usefully described as the person with dementia responding in a new or unexpected way (as discussed above), leading the carer to see them in a new way (the outcome). It can be difficult in realist theorising to categorise each element of the theory in the 'correct' position. It is possible that another researcher would suggest that the process of the carer seeing the person with dementia in a new way is the mechanism, with something else as the outcome of that process. However, I think the process of having a new or adjusted set of expectations about a person is an outcome, which could then go on to impact other interactions and activities in different ways (in realist terms – this outcome could become the context in new CMO configurations). In the original review theory, the mechanism is the perception that has changed in the carer, with the outcome being that perception as changed, which is only negligibly different. Having the person with dementia's response as the mechanism is clearer about what is actually happening, and so is more useful in considering how the outcome is generated and what implications this may have.

Following the evaluation, this theory has been refined as follows:

9. When the facilitation enables the person with dementia to 'maximise capacity' to participate in an activity outside of their usual routine (M/res), the person with dementia responds in a new or unexpected way (M/rea). Where the carer's expectations of the person with dementia are low (C), and/or others in the group highlight the person with dementia's capabilities (C), the carer sees the individual person with dementia in a new way (O) or reconsiders the activities in which people with dementia in general are capable of engaging (O).

Theory 10 – (review theory b)

From the review:

> "Professional carers only have a relationship with the person with dementia in a work context (C) but take part in a shared activity outside of the work context (M/res) and are able to get to know the person with dementia beyond dementia (M/rea), which builds their relationship"

Due to ethical approval restrictions, most professional care dyads in observations or interviews could not be included as DK could not be sure if they were funded by social care. This means that the evidence for theories about them is limited to an interview with one professional carer dyad who could be included, and descriptions of other professional carer dyads in staff interviews.

Two members of staff at site 1 described situations where a professional carer dyad did not know each other well, but came to the sessions which treated them both as equal participants. Through their participation they learnt new things about each other and shared an enjoyable experience, which built their relationship with one another. This appears to support the full CMO of theory 10.

> *…her daughter paid for a carer to spend time with her once a week I think. And this paid carer was a really not very confident young woman and at first she didn't enjoy being in the sessions being, I think she felt a little put on the spot when she was asked to give opinions or say anything. And then she kind of started coming out of her shell a little bit … And then it…over time it started becoming something that they were doing together. So she actually started requesting that her day's work, that she could work with this older woman on the days she was coming to the museum. And they started looking forward to doing it together. They both got little butterfly magnets from the museum shop that they had at home. And you could really see the bond between them building…that might just have happened over time anyway. But it felt like, it felt like it helped to provide something for them to bond around and to see each other as, I think to see each other as people in a humanising way.*
> *- Manager interview, site 1*

There was also evidence from staff interviews supporting the mechanism's resource (M/res) of the shared activity outside of a work context leading to the mechanism's reasoning (M/rea) of the dyad learning new things about each other. For example:

> *And [at] our last [site] tour, the paid carer said "oh it was really nice to see, to actually sing with him and to get to know him more as well".*
> *- Manager interview, site 5*

However, in the one interview with a professional carer and person with dementia dyad, they both stated they had not learnt new things about each other through coming to the sessions as they already knew each other well and had done a number of activities together. This person with

dementia lived in the community rather than a residential home, so the dyad had more of a one-to-one relationship. This may be different in a care home setting with a number of different staff members and residents where they do not have as much opportunity to develop a one-to-one relationship.

The evidence from the evaluation therefore refines the context – it is not solely that professional carers only have a relationship with the person with dementia in a work context, but also how well they already know each other or how much they have already engaged in social activities.

Given the evidence for this theory is limited both in this evaluation and in the review due to the lack of inclusion of professional carers, conclusions drawn can only be tentative.

The theory has therefore been refined as follows:

10. Professional carers who only have a relationship with the person with dementia in a work context with limited opportunities for shared or social activities (C), take part in a shared activity outside of the work context (M/res) and are able to get to know the person with dementia beyond dementia (M/rea), which builds their relationship.

Theory 11 – (review theory c)

From the review:

> "*Where dyads have little opportunity for meaningful/leisurely activity* (C), sharing a meaningful experience on an equal basis (M/res) means they have meaningful communications and interactions (M/rea) which helps the carer to see the person with dementia in a new way (O) and build their relationship (O)."

There was some evidence from staff interviews of dyads having new conversations and this leading to the carer seeing the person with dementia in a new way or building their relationship. However, the mechanism's resource appeared to be experiences outside of their usual routine, rather than a *meaningful* experience.

> *And I think sometimes we've seen, certainly with one daughter and mother relationship, you could see her re-visiting her mother, just sort of seeing her mother slightly differently to when she arrived. Which is quite interesting. In a good way, you know, her mum was coming out with things that she just probably would never have come out with because they just go through the normal routine every day of just doing whatever you do, you know. And by being challenged and asked different things she just sort of saw a different side of her mum. And that was quite nice.*
> *- Support staff interview, site 2*

However, the family carer interviewed said she had not learnt new things about her partner due to their longstanding relationship (which was also the reason given by the professional carer interviewed as discussed in Theory 10 above).

> *Sometimes he'll say something about what he's done in the far past and I think "was that true or not?" [laughs]. Something I didn't know. But I don't think [we've learnt anything new about each other], no not really. We've been together for 35 years so I know him pretty well.*
> *- Carer interview, site 4*

This was also something found in the review – that some family carers felt they did not learn anything new about their partner due to the length of their relationship (Lamar, 2015). It may be

that carers are more likely to learn something new about the person with dementia when they do not already know them well, for example, professional carers who have only had a short or intermittent relationship with the person with dementia, particularly if they are living in a residential home. However, the evidence from the review for this theory also included family carers, so it is not limited to those professional dyads who may have had shorter relationships. It may also be related to how in tune the carer is with the person with dementia's current identity and interests (as described in Theory 5, on carers forcing a particular kind of engagement) and how open or able they are to accept any changes in the person with dementia if they are present.

There was no evidence in the evaluation related to the hypothesised context, mainly because the two dyads who were interviewed said they did not learn anything new about each other so this could not be explored. Given the discussion above, the context may be more about how well the dyad knows each other. The hypothesised context of having little opportunity for leisurely activity may not lead to the carer to see the person with dementia in a new way if they already know them well. For example, the carer who was interviewed at site 4 discussed how she and her husband were struggling to go out and do activities after his diagnosis before coming to the museum, but she also said they did not learn new things about each other (despite all elements of the theory applying to their situation).

This theory is linked with Theory 10, examined in the previous section. Theory 11's altered context (not knowing each other well) and mechanism (sharing an experience outside of their usual routine) is similar to Theory 10's context (the professional carer only knowing the person with dementia in a work setting) and mechanism (getting to know them through sharing an activity outside of that context). Theory 11 could, therefore, supersede Theory 10, particularly as there was less evidence for Theory 10 given most professional carers could not be included in this evaluation. However, the 'work context' includes more than just caring tasks, for example the culture of the care home, professional policies, and training. For this reason, Theory 10 will not be incorporated into Theory 11, in order to highlight particular issues around participating in the session while at work.

This theory could also be linked with the Theory 7, which discusses dyads having new conversations *after* the session, as well as within. The mechanism of Theory 7 is not split into resources and reasoning, and is simply sharing an activity outside of caring routines. This is the resource component of the mechanism for Theory 11, with the reasoning component being having new communications and interactions within the session. It may be that having new interactions with each other within the session is the mechanism resource for having new things to talk about and share with others after the session. However, it may also be possible for a dyad to enjoy a session separately, so not have much interaction within a session, and still have new things to talk about afterwards, prompting each other as discussed in Theory 7, as they participated in the same session with the same people and activities. Additionally, the focus of the two theories is qualitatively different – Theory 11 is essentially about getting to know one another (either for the first time or again), whereas Theory 7 is about sharing together after the session (which may not include getting to know one another). For example, the professional carer dyad quoted in Theory 7's evidence discussed sharing after the session being a benefit of attending together, but also stated they did not learn new things about each other because they already knew each other well. This suggests the differing contexts are key. For this reason, as well as dyads not necessarily needing to interact in the session to prompt each other in sharing afterwards, the theories will remain separate programme theories.

This theory has been developed as a result of the evaluation as follows:

11. Where dyads do not know each other well, or the person with dementia has changed some of their interests or aspects of their identity (C), sharing an experience outside of their usual routine on an equal basis (M/res) means they have meaningful or new communications and interactions (M/rea) which helps the carer to see the person with dementia in a new way (O) and build their relationship (O).

Theory 12 – (review theory d)

From the review:

> "*In dyads where the carer is unsure about how best to support the person with dementia or is struggling with communication (C)*, modelling by the facilitators (M/res) means carers learn new ways to interact with the person with dementia and new strategies (M/rea), which builds relationships through improved communication and a focus on process rather than product (O)."

The context was only theorised, rather than evidenced, in the review. In this evaluation, the context appeared to be about having habitual patterns of interacting, or care homes having a particular culture so staff all work in a particular way with people.

> *…and also I think it's quite good for [professional carers] to see other people talking to their clients, because in a care home environment it's just them all the time, whereas these are completely different people coming from a completely different viewpoint talking to their clients, and I think that's good. I did notice on one or two occasions that I would start talking to the client in a certain way, and the carer then changed their way of talking to them into, not mimicking, but taking a reference from the way I was talking to them. …Not necessarily better, but just differently.*
> *- Support staff interview, site 2*

> *And then there is the issue of maybe being used to kind of helping the person quite quickly, or speaking for them a little fast, or, whereas for us as staff in the museum we might be a bit, wait a bit longer or try a few more different ways of accessing. If the person isn't very verbal there might be different ways in which we kind of enable that. Whereas there are kind of patterns already set up often within that kind of [caring relationship].*
> *- Manager-facilitator interview, site 2*

Staff interviews supported the mechanism that staff interact differently with the person with dementia, and that this may lead to changes in the way carers interact with them. For example, modelling in the session that people with dementia could speak for themselves and would be given the time needed, which could then reduce the amount carers intervened on the person with dementia's behalf within the session. They also discussed museum staff finding new ways of doing activities, and this helping the dyad to find new ways of interacting. Both of these are demonstrated in the two staff quotes above.

One manager at site 1 also suggested that the reason staff (and others in the group) are able to interact differently than family carers is because they have an emotional distance from the person with dementia that family carers often cannot have.

> *…it's not just us that model behaviour, you see other people with dementia and other carers often really patient. Well that same thing where it's so much easier to be patient with somebody that you're not connected to.*
> *- Manager interview, site 1*

However, there was also a suggestion that staff interactions do not have an impact on the way a carer interacts. Two staff members from sites 2 and 4 suggested that because there is a short period of time in the sessions (usually two hours), and many dyads have longstanding habitual patterns of interacting, staff behaviour does not change how that dyad interacts outside of the session. They also suggested that because museum staff are not trained in social work or care work, it is not the place, and not the aim, for sessions to change dyad interactions.

> *Staff member: …But I can see that particular carer does get quite frustrated with looking after [her husband]. And she does, you can see how frustrated she is and she tells him off for all sorts of things and you just feel like saying "no [carer], it's alright, perhaps go about this a different way". But I can't. I can't do that. I can't, yeah I don't feel that's my role really. I think that would change the role, my role.*
> *Interviewer: So your role isn't to intervene when you feel like someone's, the dynamic of someone's relationship is…*
> *Staff member: No, I couldn't do that. And I don't think, because I'm only with them once a month, you know, for a couple of hours. I mean, what on earth could I possibly do that would make any difference at all. But it would need to be somebody from within the community services really.*
> *- Support staff interview, site 4*

"Habitual patterns of interacting" was suggested as the context for carers learning new ways to interact, but also as the context for why carers *do not* interact in new ways. It may be that, in some cases, the resources do not have enough leverage to overcome the context of habitual patterns. Alternatively, it may be that this suggested context is not specific enough to be useful. It may be, similarly to theories 10 and 11, the difference is between those dyads with longstanding relationships (such as spouses or professional carers who have a one-to-one relationship with the person with dementia) and those with more recent or intermittent relationships (such as professional carers in a residential home). The original theorised context may also apply within this. For example, if a person with dementia and a carer do not know each other well, the carer may not yet know the best way to support them, so the facilitators and activities give them new ideas of ways to interact and this helps to build their relationship.

There was no direct evidence regarding the outcome of theory 12 in the evaluation, so the evidence for this outcome is mainly drawn from the review. However, the examples staff members gave were around a shift in process, such as giving the person with dementia more time to speak. This may support the review findings in suggesting carers were less focused on, for example, giving the 'correct answer' (product), and more on the process of the person with dementia finding their own answer.

It should be noted, however, that the evidence from the evaluation is limited by only coming from staff interviews, and not from carers saying they are doing anything differently, nor from observations of carers seemingly trying out ways of interacting they witnessed in the session.

The review theory has therefore been revised as follows:

12. In dyads where the carer does not know the person with dementia well (C), is unsure how to support the person with dementia (C), or has fallen into habitual patterns of interacting (C), staff interacting with the person with dementia in a new way (M/res) means carers learn new ways to interact with them and new strategies (M/rea), which builds their relationship through improved communication and a focus on process rather than product (O).

Theory 13 – (review theory e)

From the review:

> "*Where the person with dementia is anxious about new activities (C)*, the carer reassuring or modelling taking part in a shared activity (M/res) helps the person with dementia to feel comfortable/able to take part (M/rea), so they are able to participate without anxiety (O)."

There was evidence in session observations at sites 1, 4 and 6, interviews with staff at sites 1, 2, 3 and 4, and people with dementia at site 4, that carers provide reassurance to the person with dementia. And that this reassurance leads to the person with dementia participating in the session or feeling more comfortable in the session. There were three main ways carers provided reassurance in sessions:

a. Through their physical presence or proximity to the person with dementia

This could be simply the carer's presence in the session without them necessarily actively doing anything, being there so the person with dementia does not have to go to a new environment or group alone, or doing the activity alongside the person with dementia.

> *During the tour they mostly walked/sat together. Occasionally [the person with dementia] would look unsure, then stretch out her hand and touch [carer] (his back or his hand) and visibly relax.*
> *- Fieldnotes, site 6*

> *Person with dementia: I couldn't come by myself [the first time].*
> *Carer: I don't think you'd have ever come by yourself.*
> *Person with dementia: No, absolutely not.*
> *Carer: But I mean, I think you would now. …[person with dementia] lives literally over the road so I think, in theory, you would come. If I said "oh I can't come but it's this morning"*
> *Person with dementia: Oh no I would come now, definitely.*
> *Carer: Exactly, now she knows people.*

- Professional carer & person with dementia interview, site 4

b. Physical reassurance

Some carers demonstrated reassurance through physical gestures.

> *Occasionally [carer] touched [person with dementia]'s back and stroked it.*
> *- Fieldnotes, site 1*

c. Answering anxious questions and/or re-explaining what is happening

A few people with dementia repeated the same questions to their carer, such as repeatedly asking for the time or where they were going, and the carer responded by answering their question or explaining the session and the activity they were doing.

> *Like for example there was one of the people who came to that session, he, I think he went to say "oh I'm going to go" or "it's time to go" or something. And she said, she was from the Alzheimer's Society I think, and she said "oh aren't we going to do this" or "we're not going to go yet because of this". And she distracted him kind of from what his thoughts were, so that he'd come back to the activity that was taking place.*
> *- Manager-facilitator interview, site 3*

> *In the next part of the gallery, [person with dementia] asks "where are we going?" And [carer, her daughter] said "we're just gently walking around". [Person with dementia] said*

"oh okay". Throughout the session there seems to be lots of small reassurances like this from [carer] to [person with dementia].
- Fieldnotes, site 1

People with dementia appeared to be anxious in a range of ways within, or about, sessions (from anxiety about initially attending, to a more general anxiety), and the people with dementia who were anxious within sessions included those who had attended previously (so the activity or group was not new to them). This suggests the context may also include new groups of people as well as new activities, and generalised anxiety, as this can be a common experience of people with dementia (Seignourel et al., 2008).

There were a number of ways carers had responsibility for the person with dementia they support, and reassurance was just one of them. It is therefore important to consider why this particular way of supporting people with dementia has its own theory separate from any overarching theories about caring responsibilities in sessions. Aside from this being a well-supported theory, reassurance-giving appears to be one of the 'responsibilities' that museum staff can never fully take on behalf of the carer. In sessions, people with dementia always looked to the carer, not a member of staff, for reassurance, which is unsurprising given that they do not know the staff as well as their carer. If the person with dementia needs the carer to provide reassurance, particularly if they need physical reassurance or close physical proximity, this has implications for the function of the session for the carer (as described in the section on Theories 1 to 3). If the carer wants more traditional respite, they are not able to have this and be separate in the session if the person with dementia needs them to be physically close, as staff cannot give the person with dementia the reassurance they need to be comfortable. It therefore seems important to highlight this type of caring responsibility separately, and link it to session function.

Though only one study in the review supported this theory, it was generally well supported in the evaluation, and has been revised as follows:

13. Where the person with dementia has anxiety, or is anxious about new activities or new people (C), the carer reassuring or modelling taking part in a shared activity (M/res) helps the person with dementia to feel comfortable/able to take part (M/rea), so the person with dementia is able to participate without anxiety (O), but the carer cannot be separate within the session (O)

Theory 14 – (review theory f)

From the review:

> "*Where the dyad's home interactions are mainly around caring (C)*, enabling facilitation in a non-medical setting in which the carer has no caring responsibilities (M/res) means the dyad can enjoy the activity together on an equal basis (M/rea), which leads to the dyad experiencing shared respite from dementia roles/life (O), which also helps to strengthen their relationship (O)."

Five staff interviews at sites 1, 3, 4, and 5 suggested that, rather than it simply being a 'non-medical setting', it is the sessions not being about dementia, talking about difficulties, or reminiscence, which was the mechanism for respite. The evidence for this part of the theory from the review came from three papers, with only one directly noting the 'non-medical' setting (Johnson, Culverwell, Hulbert, Robertson, & Camic, 2017). The others stated that it was about it being a 'normalised' public space (McGuigan, Legget, & Horsburgh, 2015), or that it was doing an activity that was not about medical care (Camic, Tischler, & Pearman, 2014). Four other papers (reporting on three studies) also

supported the idea that the activity not being about dementia was linked to shared respite or enjoyment (Baker, 2014; Burnside et al., 2017; Camic et al., 2016; Flatt et al., 2015). It may therefore be more specific (and so more useful in terms of thinking through implications of theories) to re-write this part of the mechanism as being an activity not about dementia, in a community space. This also integrates Camic et al.'s (2016) grounded theory (included in the review) which suggests an important aspect of these museum programmes is the museum setting as a 'valued place'.

> *…from the point of view of what's good for everybody is that sometimes what makes you feel good isn't talking about your problems. I felt like it was helping people to feel like themselves.*
> *- Manager interview, site 1*

> *…it doesn't matter that they've got dementia, they're both new to this, and it's a new experience. And it's not about reminiscence, it's not about looking at the past, their past, or trying to remember things, they don't have to, there's no work involved.*
> *- Support staff interview, site 4*

There was also evidence for an enabling facilitation (mechanism resource) leading to equalising between the person with dementia and carer (mechanism reasoning) from staff interviews at sites 2 and 6. Some staff at sites 1, 2, 3, and 4 felt that the activity being new to both the person with dementia and the carer also helped to equalise.

> *[Support staff] said she felt there was a high level of engagement from both the person with dementia and the carers, and it felt like a happy session. She said it felt like everyone was able to get involved at a level they felt able to*
> *- Fieldnotes, site 2*

> *It's a shared new experience for a lot of them. Because a lot of people say "oh we've been to the museum before" but they've probably never been on a tour before, and certainly not a tour that's specifically for them, it's for them. And I think there is an element of it being special for everyone, and it being an experience for everyone.*
> *- Manager interview, site 5*

Staff members at site 2 thought staff also taking part in the activities helped to equalise between participants because it modelled that every attendee is an equal part of the session and that facilitators do not differentiate by role, which helps carers who are unsure if they can step out of their caring role to participate.

> *It's the same with the volunteers and the [support] staff, everybody in the group. And that's how you create a kind of collective kind of community feel in a group, I guess. Where people all feel welcome and there's not hierarchy of "oh I'm this and you're that". So I think that's quite important.*
> *- Manager-facilitator interview, site 2*

One staff member at site 1 and four at site 6 highlighted the fact they did not make a distinction between who was a carer and who was a person with dementia, and often did not know what role participants had as it was never discussed.

> *And I can't really tell the difference between who is the carer and who is the dementia patient, because I don't make those connections, you know, they come in as a couple or they wander in, you know, or I wander in after they've arrived, and I have no idea who's the dementia patient and who's the carer.*

- Facilitator interview, site 6

However, although some staff members said they did not distinguish between carers and people with dementia, many of those same staff members did feel the carer had caring responsibilities in the group, so they did make a distinction, even if they felt it did not impact their facilitation style. For example, one of the facilitators at site 6 said not knowing who in the group has dementia helped to equalise, but also suggested in another part of their interview that carers were primarily included for the responsibility they could take.

> *No, we're not told [who has dementia] and we just put on our name badges. So it's only in conversation that you might realise there is a bit of a problem perhaps. Which is good, because then when you're sort of, you're embracing everybody as a group.*
>
> *…because [the professional carer] almost needed to be reminded that she was here to help the person rather than to sort of taking off herself.*
>
> *- Facilitator interview, site 6*

This 'equalising' appears to be the key part of the mechanism, rather than enjoyment. The review studies which led to the development of theory 14 were mixed in terms of either suggesting it was enjoying the activity together which led to shared respite, or that it was participating as equals. As shared respite is about stepping outside of caring/cared-for roles, it would make sense that this is about an equalising or, in other words, not participating differently according to your caring role of being a carer or cared-for.

Three staff members at sites 2, 4, and 6 suggested it being a non-caring situation and activity was a mechanism for respite, implying the lack of caring responsibility is important.

> *And that grandmother and granddaughter, it was nice to spend that time with her grandma but not in a situation where she was caring for her. So it was a very, it's a very different scenario that people are in and people, there were quite a few cases of people really saying that they really enjoyed that and they really valued that.*
> *- Manager-facilitator interview, site 2*

However, as discussed in Theories 1 to 3, the interaction between caring responsibilities and respite is complex, and not the same for every dyad. Some carers appear to feel respite at the same time as having a number of caring responsibilities, whereas others need to have few, to no, responsibilities to feel respite in the session. Additionally, carers always have *some* responsibility for the people with dementia in the group. There are limits to the support staff are able to offer the person with dementia, and carers are often included *because* they can be responsible. This suggests that the mechanism of 'the carer has no caring responsibilities' is not accurate. Additionally, even where the person with dementia needs a high level of support from the carer, the session always takes *some* responsibility from the carer simply through the structure of the session, which takes care of the content and provides some support for the process. As described in the new theories on session function, the higher staffing levels in these sessions also help to minimise what carers need to do, to an extent.

> *Because it's a facilitator-led session with lots of support you can kind of, I mean I hope, they can kind of just let go because things are being done, they're just going along with the session and whatever's going to happen and it's led by someone and what we're going to do is all mapped out.*
> *- Manager interview, site 4*

Given that different dyads use the session in different ways, it may be that the resource is not about a lack of caring responsibilities, but about the carer only having the level of responsibilities they want or are comfortable with, combined with some support from staff and the session structure.

This link to session function may also suggest the context is not only about a dyad's interactions being mainly about caring, but also that they want to do something together. Those who want to enjoy the session separately are unlikely to experience shared respite, whether they spend the session together or not.

Therefore the theory has been re-worded as follows:

14. Where the dyad's home interactions are mainly around caring and they want to spend time together (C), enabling facilitation of an activity not about dementia in a community setting, in which the carer only has to provide the level of care for the person with dementia that they want to (M/res), means the dyad can participate on an equal basis (M/rea), which leads to the dyad experiencing shared respite from dementia/caring roles (O), which also helps strengthen their relationship (O).

Theory 15 – (review theory g)

From the review:

> "Shared respite (C) means the dyad can interact and communicate outside of dementia roles (M), which builds their relationship (O) and also feeds back into enjoying the activity together (O)."

There was a view that shared respite could be relationship-building in staff interviews at sites 1 and 5.

> *…to help try and reframe your relationship about something on an equal level that's not around caring or being cared for, that you can, I feel like sometimes little things can tilt the balance of a relationship and it doesn't necessarily have to be over time that…any relationship can get out of balance in different ways and so I feel like it would be great if just…a morning at the museum could help to rebalance your relationship.*
> *- Manager interview, site 1*

> *It's also about [the professional carer], "did you have, were you a rebel when you were younger?" Because that, again, that kind of brings out similarities between them and they can realise that they do have these shared life experiences even if they weren't at the same time in their past.*
> *- Manager interview, site 5*

And there was some evidence (from staff interviews at sites 1, 3, 4, and 6) for having conversations and doing activities that were not about dementia or their caring roles, leading to a shared enjoyable experience. Enjoying the activity together (as distinct from needing to be together due to the person with dementia's needs) was well-supported as an outcome. As described in Theories 1-3, evidence was found in fieldnotes at all six museum sites and from descriptions in six interviews (one with a professional carer-person with dementia dyad at site 4, and five with staff at sites 1, 2, 4, and 6).

> *[Person with dementia] also told me she likes to have a joke after laughing with [her professional carer] about a painting in which the model would have been sitting with a bottle of water in her cleavage in order for the painter to get the flowers right.*
> *- Fieldnotes, site 2*

...whether it's making music or drama, then it's really nice for them to share a moment, for me to see them share a moment with their person they care for, perhaps doing something they would never normally do together.
- Manager interview, site 4

Some evaluation forms from site 5 also focused on shared enjoyable experiences as a positive from the session, suggesting it was seen as a key outcome for those carers.

So actually, to be honest, most of them do focus on what they liked, but usually it is those shared experiences, so like, in some of our evaluations we've had "we loved…", especially at [site], "we loved singing". And it's always WE loved singing.
- Manager interview, site 5

As discussed in the previous section on Theory 14, the relationship between shared respite and caring responsibilities is complex. This means the relationship between shared respite and shared enjoyment is also complex because of the individual differences in how caring responsibilities impact on enjoyment. Shared respite and shared enjoyment are closely related concepts, but not the same – shared respite is about the focus of the relationship not being defined by dementia, whereas shared enjoyment is taking pleasure in the activity together. In the review it was suggested that they are mutually reinforcing processes – shared enjoyment contributing to shared respite, and shared respite further enabling them to enjoy the activity together. Although this makes conceptual sense, this link is now less clear at a theory level as 'enjoying together' was removed from theory 14 in favour of 'participating equally'. It may be that theory 15 is not separate from theory 14, but a CMO which details the process between the two outcomes of theory 14 (shared respite and relationship building).

No changes were made to theory (g) as a result of the evaluation.

15. Shared respite (C) means the dyad can interact and communicate outside of dementia roles (M), which builds their relationship (O) and also feeds back into enjoying the activity together (O).

Theories 16-18 – (review theory h)

From the review:

"The participatory activity with a 'product' e.g. artwork/verbal contribution (M/res), *in the context of high carer expectations or a dyad struggling to accept dementia (C)*, means the carer compares the person with dementia's current abilities to their former abilities or negatively with other people with dementia in the group (M/rea), which leads to the group highlighting losses or reinforcing limitations (O)."

All of the evidence in the evaluation for this theory related to family carers, rather than professional carers. This evaluation could not directly include the majority of professional carers due to ethics permissions, which may be why the evidence for them is missing here. However, as discussed in Theory 11, family carers may have had longer relationships, and more emotional history, with the person with dementia they support. This means theories related to that history or emotional connection may be more likely to apply to them. The five papers in the review which supported the development of theory (h) included a mix of family and professional carers, or did not specify the dyad type, which is why it was not specified in the review theory. However, as it concerns having a relationship history, it makes sense that this theory would apply more to family carers. The way those review papers report losses being highlighted implies, or directly states, a personal history

between the person with dementia and the carer. For example, Humphrey et al. (2017) discussed some carers feeling discouraged that the person with dementia's artwork was less skilled than that they had produced in the past. This personal history suggests these results apply to family, rather than professional, carers. As this is also where the evidence lies in the evaluation, theory (h) will be made more specific to family carers.

There was evidence from six staff interviews at sites 1, 2 and, 4 that losses can be highlighted in the sessions.

> *Sometimes the carers have found it hard during the sessions, and we've had quite a few instances where the carers will break down and cry because it's just so hard to watch their grandmother or wife in that situation. I guess, well who knows what they're thinking, but, you know, potential of what might have been or whatever. But I think that's quite hard.*
> *- Support staff interview, site 2*

There was also some evidence of carers comparing the person with dementia's current abilities to their former abilities in interviews at sites 1, 2, and 4.

> *And [the person with dementia] was finding [the art activity] really exciting and stimulating and [his wife] was going "oh that's not how he used to. Oh his stuff used to be really good and that's not what he used to do." It was really quite sad.*
> *- Facilitator interview, site 1*

The review suggested that carers negatively comparing the person with dementia they support with others in the group was a mechanism (negatively meaning that the person with dementia has more advanced dementia or greater difficulties than others in the group). Five interviews with staff, people with dementia, and carers at sites 3 and 4 suggested there is also a mechanism around comparing the person with dementia with others in the group *positively*. That is, the person with dementia is more mildly affected by their dementia than others in the group, and seeing people with dementia who have greater difficulties highlights a possible negative future.

> *Well the only, yeah the bad thing [about the programme] would be seeing somebody gradually decline and seeing the difficulty they have.*
> *- Carer interview, site 4*

> *And she didn't volunteer again. And I spoke to her and I said "did you enjoy it?" And she said "yeah yeah I did enjoy it", but I could tell there was something going on there…And it was because she personally has been affected by dementia that she found it a bit much…she also said she was thinking of maybe her possible future [as a carer].*
> *- Manager-facilitator interview, site 3*

However, one member of staff suggested this happens less in their museum sessions than in carers' groups they ran in the community because people stop attending the museum as their dementia progresses:

> *I've run carers' groups and carers' groups are quite worrying like that, when someone's clearly looking after somebody who's really way down the road, and they think "oh god that's going to be me". But that hasn't really happened in this context because everybody's got quite early stages. And I think when they are deteriorating and that they're not really managing very well, they don't come back.*
> *- Support staff interview, site 4*

Descriptions in interviews also suggested that the resource is not just about the activity having a 'product', but that it is an activity outside of their usual routine which means the person with dementia is asked to do things they may have stopped doing in their day-to-day life. For example:

> *There was [wife] and [husband] who came today. The first session they came to, we asked them to write some, write their name on something, and [husband] couldn't write his name and [wife] didn't know he couldn't write. And I think that really upset her. Because she didn't know, so that was quite sad for her.*
> *- Support staff interview, site 4*

The carer feeling respite from their caring role could also be a context for the programme highlighting losses for them. A family carer being outside of their caring role, which they may not have felt for some time, makes them realise there are activities they miss which they used to enjoy, highlighting the carer's own losses. Carer respite is thus not always a positive experience if it also highlights these losses.

> *[Support staff] discussed how [carer] spoke to her about how she had suddenly realised a few days ago that she no longer does anything creative for her – everything is taken up with the care of her husband. [Support staff] said [carer] told her it had been a very emotional realisation and that she seemed to be feeling a little sad about it when she was talking to her.*
> *- Fieldnotes, site 2*

For one carer, however, this way of highlighting losses actually became a positive context. After these losses were highlighted and she was upset, she realised she needed to find other activities that were for her, and then sought out and joined another group (which did not include her spouse).

> *The wife had been really creative and she actually got really emotional in the session because I think she just realised how she didn't have creativity in her life anymore and that, that reminded her of that. And that, she then got in touch with a craft group near [city]. She managed to get that as part of her regular week, to have something else going on.*
> *- Manager interview, site 1*

However, much like theories 26 and 28 on opening up the museum, this likely depends on the carer's capacity to join other groups, whether through finances or the person with dementia having other sources of support while the carer attends, so this realisation could also be negative if they then do not have this capacity.

There were also no results in the evaluation which related to the hypothesised context of this theory, so this will remain italicised for transparency.

The review theory has been revised, and two new theories developed, as follows:

16. The participatory activity with a 'product' that is outside of their daily routine (M/res), *in the context of high carer expectations or a dyad struggling to accept dementia (C),* means the family carer compares the person with dementia's current abilities to their former abilities or with other people with dementia in the group (M/rea), which leads to the group highlighting losses or reinforcing limitations (O).

17. Where the family carer feels respite in the session (C), they realise they are missing other activities they enjoy (M), and this highlights personal losses for the carer (O).

18. When personal losses are highlighted for the family carer (C), they realise they need to find activities just for them and seek them out (M), and then join other groups without the person with dementia (O) if they have the capacity to do so.

The outcome of Theory 17 becomes the context for Theory 18.

Theory 19 – (review theory i)

From review:

> "*Where the carer has high expectations of the person with dementia (C)*, or the group highlights losses (C), the carer does not perceive the person with dementia to have achieved competency (M), so the carer does not feel respite (O) and does not see the person with dementia in a new way (O)."

As discussed in the section on Theory 9, the language of 'competency' is judgemental and will be changed to 'capabilities'. Like theories 16-18, the evidence for this theory from the evaluation only relates to family carers, and it was not always clear in the review to which dyad type the evidence was referring. However, as discussed in the section on Theory 11, family carers may have had longer and more emotionally laden relationships with the person with dementia they support, so theories related to that history or emotional connection are more likely to apply to them.

Two staff members describing the same dyad at site 1 suggested evidence for the full CMO. In this case, others in the group highlighting the person with dementia's capabilities (as per theory 9), did not have enough leverage to overcome the carer comparing the person with dementia's current to past abilities, so she did not see him in a new way or from a new perspective.

> *Facilitator: And [the person with dementia] was finding [the art activity] really exciting and stimulating and [his wife] was going "oh that's not how he used to. Oh his stuff used to be really good and that's not what he used to do." It was really quite sad.*
> *Interview: What do you do in that situation?*
> *Facilitator: Oh well I suppose for me I say "Oh I really like that. I really like the thing that he's done", but, you know, obviously I've not seen the things he used to do. But I just sort of said "I really like it", but it didn't change her opinion. To her it was just a mess.*
> *- Facilitator interview, site 1*

As well as the mechanism being the carer not perceiving the person with dementia's capabilities, the evaluation also suggested that the carer feeling upset following losses being highlighted may be a mechanism. It may be that this upset feeling then contributes to the carer not feeling respite. In the examples given for highlighting losses in the section on theories 16-18, all involved the carer showing upset:

> *…it had been a very emotional realisation*
> *- Fieldnotes, site 2*

> *…we've had quite a few instances where the carers will break down and cry*
> *- Support staff interview, site 2*

> *…I think that really upset her…that was quite sad for her*
> *- Support staff interview, site 4*

> *…she actually got really emotional in the session*
> *- Manager interview, site 1*

Like Theory 16, there was no evidence in the evaluation related to the hypothesised context of the carer having high expectations.

The theory was revised as follows:

19. *Where the family carer has high expectations of the person with dementia (C)* or the group highlights losses (C), the carer does not perceive the person with dementia's capabilities or feels upset (M), so the carer does not feel respite (O) and does not see the person with dementia in a new way (O).

Theory 20 – (review theory j)

From the review:

> "When the carer has caring responsibilities in the group (C), the dyad cannot enjoy on a completely equal basis (M) so the carer does not get respite (O) and feels increased stress (O). (Not an on/off, but 'dimmer switch' programme theory as per Dalkin et al. (2015). Also may be about 'perception' of responsibilities, as unlikely to have none at all)."

There was some evidence for stress related to caring responsibilities, especially the logistics of getting to a session. However, this is limited to staff perceptions, rather than carers describing sources of stress themselves.

> *…because bringing someone to the museum, it's a new thing for [the carer] as well, coming, negotiating transport, finding the entrance to this place is impossible, and things like that can add so much stress to a person.*
> *- Manager interview, site 5*

> *I think sometimes people can be a bit stressed [at the start of the session] because it can be quite an early start to get here, or they might have had a difficult night, or be having a bad week, or the weather's been bad, or traffic's been bad.*
> *- Manager interview, site 4*

There was also some evidence that having higher caring responsibilities in the session meant the carer could not engage with the activity, but they were able to engage when they had less caring responsibilities. Carers often enjoyed the session through the activity and setting itself, so if the carer cannot engage with those things due to the level of responsibilities they have, it may be less likely they would be able to enjoy the session. The following quote from the manager at site 3 describes why she thinks carers engage less with the tour in the first half of the session, and more with the object-handling in the second. She suggests the carer was only able to engage with the session's activities when they had fewer responsibilities.

> *…but sometimes with the tour, [the carers] can't really engage in what we're doing because they're trying to kind of make sure the people they're looking after are okay. So I think sitting down at the table really does help them also engage, because that's the moment where actually that person's not going to wander off, they know that person's probably mostly comfortable sitting down or whatever.*
> *- Manager-facilitator interview, site 3*

Relatedly, there was also some evidence linked to session function (as described in evidence for theories 1-3) that having more responsibilities in the group means less enjoyment for the carer. When the carer wants to use the session as more traditional respite, but cannot be separate in the session due to needing to support the person with dementia, they enjoy it less. This was most

evident when the person with dementia's needs increased and the carer had to change from being separate in the session to assume more of a caring role. For example, as described in the section on theories 1-3, at site 1, Robert (carer) liked to enjoy the session on his own, but increasingly needed to support his wife, Stella, and often expressed frustration and immediately moved to be by himself after supporting her.

Carers always have at least some responsibility for the person with dementia in the group, and are usually included because they can take that responsibility. It is therefore unlikely that the context is simply that 'the carer has responsibilities' because they always do. This was accounted for in the review to some extent by suggesting it was a 'dimmer switch' rather than an on/off process, but it was unclear what specifically it was about responsibilities that lead to the carer not being able to feel respite or enjoy the session.

As discussed in theory 14, it is likely the context is linked to session function and the match (or mismatch) between how the carer *wants* to use the session and how they are *able* to use the session. This is dependent on the expectations of the museum staff, the level of support the person with dementia needs, and whether this support is within the limits of responsibility staff can take.

Theories 1-3 on session function will also link with this theory, as it is not about having caring responsibilities per se, but the match in session function for that carer and the person with dementia's needs. However, there are occasions when the level of the person with dementia's needs, or the staff's expectations of the carer and their role in the session, means the level of responsibility prevents the carer from being able to engage fully, or almost entirely. Theory (j) has been refined to reflect this:

20. When the carer has high levels of caring responsibilities in the session (C), they cannot engage with the activity (M), so they do not feel respite or enjoy the activities (O).

Theory 21 & 22 – (review theory k)

From the review:

> "Anxious carers (C) intervene on the person with dementia's behalf (M), so the person with dementia cannot engage fully (O) and the carer has no respite (O)."

There was evidence in fieldnotes from sites 1 and 4, and staff interviews at sites 1, 2, and 3, of carers intervening on the person with dementia's behalf.

> *As we were finishing off our drinks, the facilitator went around and asked everyone to say their names. Two carers said the name of the person with dementia they had come with, and when it was the person with dementia's turn they didn't say anything (both said "this is X, and I'm X").*
> *- Fieldnotes, site 4a*

> *Sometimes you feel like the carer speaks for them, that's quite sort of an obvious one. And when [the people with dementia] do start talking they jump in and predict what they're going to say. I think also there are quite long silences often which [carers] feel awkward about…*
> *- Support staff interview, site 2*

However, there was no direct evidence related to the impact of that (the theory outcomes) as neither of the people with dementia interviewed had carers who had intervened during the session. Evidence for the outcomes of this theory is therefore based on the review.

Additionally, in observations, it was difficult to determine if, when carers were intervening, it was unnecessary or actually a supportive practice. One thing some carers do is translate for the person with dementia, which means re-explaining what the facilitator has said in a way that makes sense to them or if they are hard of hearing. This translating can also work the other way around, from the person with dementia to the group, if the person with dementia has a particular way of communicating that others who do not know them well may find difficult to understand. For example:

> *[Volunteer] said it was good having the carers there as sometimes [person with dementia] would say something that's difficult to understand, and [her carer] would know what she said and could share it with the group.*
> *- Fieldnotes, site 2*

In this case, the carer speaking for the person with dementia is supportive, not an unnecessary intervention. Relatedly, there were times in observed sessions where the person with dementia struggled to answer, either because they seemed to find it difficult talking in groups or because they had word-finding difficulties that frustrated them. The carer, bringing that knowledge of the person's needs, answered for them as a supportive practice, not an unnecessary one.

> *I asked [person with dementia] [if she would like tea or coffee] and she paused and said "tea or coffee". I asked again which she would like, and she replied "coffee" and turned away. [Her carer] said [person with dementia] would like it with just a little bit of milk and that she would have the same, then turned back to talk to [person with dementia].*
> *- Fieldnotes, site 1*

> *…the person who came alone answered [questions to the group] most often, but...[carer, son] also answered (rarely [his father] also answered, but this was usually directed to his son who then relayed it to the facilitator).*
> *- Fieldnotes, site 5*

There were a number of possible reasons highlighted in the evaluation why carers may be anxious in a session, including:

a. The museum environment

Carers may be anxious as the museum is a new or unfamiliar environment to them, and/or can be perceived as a formal or intellectual place. This means carers are anxious themselves being in a new environment, but also about whether the person with dementia's behaviour will be 'inappropriate' in that setting.

> *I think quite often [the carers] are not necessarily people that have been to the museum on a regular basis anyway, so there's that little bit of the museum being a slightly intellectually superior place, and they're coming in very much on the back foot with a relative with dementia, so you know, not only a lack of education but also in a slightly vulnerable position in that they've got somebody with them that perhaps, you know, might be inappropriate.*
> *- Support staff interview, site 2*

b. The responsibility they hold for the person with dementia

The carer ultimately has responsibility for the person with dementia in the session and this can be stressful. As well as being unsure how the person with dementia will respond or cope with a new physical environment, this also includes issues like a recent decline in the person with dementia's health.

[Carer] asked [person with dementia] a few times throughout whether she was okay. I assume this is due to her heart attack on Monday, as I haven't noticed him doing this in previous sessions.
- Fieldnotes, site 1

I'm thinking of one particular couple where she definitely kind of manoeuvred him around the gallery I guess. And he was walking, but she was kind of like "oh step back from here" and that kind of thing.
- Manager-facilitator interview, site 3

c. Stressors in their life outside of sessions

Carers also have stressors in their life outside of the session which may or may not be related to their caring role, which they then may bring into the session as a generally anxious feeling or mood.

…carers perhaps bringing stresses of their life into the room, which is fine and understandable and everyone does that. And they might be tired or had a bad night or not particularly want to get involved, which is fine.
- Manager interview, site 4

The review theory has not been altered as a result of the evaluation, but a new complementary theory has been added about intervention as a supportive practice:

21. Anxious carers (C) intervene on the person with dementia's behalf (M), so the person with dementia cannot engage fully (O) and the carer has no respite (O).

22. Where the person with dementia struggles with speaking or finds it stressful (C), the carer answers for them in the group or translates their answers for others (M), so the person with dementia is able to engage in a way which is comfortable for them (O).

Theory 23 – (review theory l)

From the review:

"Professional carer-person with dementia dyads have only had a caring/work relationship and find it harder to step outside of caring roles (C), so the carer intervenes on the person with dementia's behalf (M), and the person with dementia cannot engage fully (O)."

As the majority of professional carers could not be included in observations or interviews due to ethical approval restrictions, evidence for theories about them is limited to descriptions in staff interviews or staff's perceptions about them.

Two staff members at site 2 suggested professional carers find it harder to step out of their caring roles, and that this leads them to intervene on behalf of, or speak for, the person with dementia more quickly.

And then there is also the issue of [professional carers] maybe being used to helping the person quite quickly, or speaking for them a little fast
- Manager-facilitator interview, site 2

However, as discussed in Theory 22, it is possible some of this early or 'unnecessary' intervention is actually a supportive practice related to the person with dementia's needs.

There was a new context suggested by three staff interviews at sites 4, 5, and 6 about how they perceive professional carers differently to family carers. These staff members saw professional

carers as only being present for their caring responsibilities, and not as having a dual role of caring responsibilities and also enjoying it for themselves, which they did see family carers as having. This was not site-specific, as different staff members at the same site varied in their view of the role of professional carers in the session. For example, in contrast to a support staff member at site 4, the manager at site 4 said professional carers do have a dual role:

> *…we did have one [professional] carer who seemed to enjoy far more than her patient, if you like, if that's a word, or client. And that was rather funny, because she almost needed to be reminded that she was here to help the person rather than to sort of taking off herself.*
> *- Facilitator interview, site 6*

> *Well, if they're paid, it's very different to if they're not paid, I think. There's been times when a paid person, who might not have ever been to [site] before, might be more interested in looking at the [site] than actually doing what they're paid to do, you know, supporting the person with dementia. That's happened a few times.*
> *– Support staff interview, site 4*

> *But, no, paid carers generally it's the same. And again they're having a nice day out as well, and for most of them quite a change from their regular routine. And they themselves might not have ever come to the [site] or learnt about that thing or enjoyed a session that's more interactive or led by someone in that way. So, no I can't say I ever notice any difference [between professional and family carers].*
> *- Manager interview, site 4*

It is possible that the professional carers would step out of their caring role, but pick up on this staff perception because of how it may then cause staff to treat them differently than family carers. This then discourages them from actively participating as an equal member of the session.

Two staff members from sites 2 and 3 discussed how it can be more difficult to encourage professional carers to participate in the session as an active participant because they are attending as part of their work day. The professional carers then find it harder to step out of, or expand, that role to their own enjoyment. This means the outcome for this theory is not just about the person with dementia's engagement, but it is also preventing the carer's engagement (and consequent relationship-building within the dyad).

> *Interviewer: Are there ever any times when it's more challenging to do that, to get everyone participating and on a kind of equal basis?*
> *Facilitator: Yeah I think there are times when, and I think it's more apparent with carers who that's their job, that sometimes there's the kind of balance of the participation can get muddied somehow, or that there is an imbalance.*
> *- Manager-facilitator interview, site 2*

The review theory has therefore been refined as follows:

23. Professional carer-person with dementia dyads have only had a caring/work relationship so find it harder to step out of caring roles (C), or museum staff perceive professional carers to only have a caring, and less participatory, role (C). This means the carer intervenes on the person with dementia's behalf or does not participate themselves (M), and the person with dementia and/or professional carer cannot engage fully (O) or gain potential relationship-building outcomes (O).

Theory 24 – (review theory m)

From the review:

> "*People with dementia may find it more difficult to speak up in a group setting (C)*, so where there is poor facilitation (M/res) carers dominate conversations (M/rea), so the person with dementia cannot engage and do not have a sense of belonging in the group and cannot gain potential positive outcomes (O)."

The new alternate theory developed on carers speaking for the person with dementia as a supportive practice, rather than an unnecessary intervention (Theory 22), also applies here as an alternate theory.

There was evidence of carers being more involved in group conversations than the people with dementia in observations of sessions at all sites except site 4. In most sessions, general questions to the group were answered by carers, not people with dementia

> *The tour was more passive than previous tours – it was more of a 'lecture-style' but with a few questions to the participants. Questions to the group were answered by carers and one person with dementia (Anna). When [facilitator] asked if anyone had any questions for him, the questions all came from carers, and included a group discussion about the cost of the art between some of the carers and [facilitator]. Anna was the only person with dementia who joined in any group discussions.*
> *- Fieldnotes, site 1*

However, there were a few sessions where general questions were also answered by people with dementia. It may be that this depends on how the dementia impacts the person or the severity of their dementia. For example, word-finding difficulties are common across different types of dementia in its early stages (Klimova & Kuca, 2016), and this may impact how easily a person is able to contribute to a group discussion if they need more time to find the words they want to say. Dementia and its symptoms progress at different rates for different people, so even if two people with dementia in the group both have word-finding difficulties, one may need a great deal of space and time to contribute verbally to a discussion, or not be able to contribute in that way at all, but another may be able to find their words quickly enough to participate.

> *During this talk, other people [with dementia] in the group made spontaneous comments about what [facilitator] was talking about it. No carers did.*
> *- Fieldnotes, site 3*

Staff interviews suggested the importance of facilitation in whether, and how, carers or people with dementia were involved in group conversations. For example, a facilitator at site 1 suggested it was important to give people with dementia space in the conversation which then enables them to contribute. This was also seen at session observations with the same facilitator at site 1, where people with dementia responded when brought into the conversation individually, but only carers were involved in the conversation at other times when they were not.

> *I think you do have to, yeah, maybe give people more time, be very conscious of giving people more time, and stop being that person who has to fill a gap, a silence, with chatter of any kind.*
> *- Facilitator interview, site 1*

> *[Facilitator] asks [person with dementia] directly what she thinks, and [person with dementia] says she wouldn't want the painting in her house because it reminds her of places*

she went when she used to be a nurse. [Carer] talks to [facilitator] about his preferred piece so far. Note – though she will sometimes, [person with dementia] makes far fewer spontaneous comments when not asked directly, but does always have something to say when she is asked.
- Fieldnotes, site 1

However, one facilitator at site 6 suggested this was not a good method for involving people with dementia in conversations as direct questions can be more anxiety-provoking for some, which actually makes it harder for them to be involved. It is therefore important to note that not all facilitation techniques work for all people.

And because I also do recall, with my mother, the last thing that was a red rag to a bull if you asked a direct question. Because there's that concern of "golly I'm being asked a question here, I know I don't know the answer" and then getting upset or getting angry or confused, and so one has to phrase things, the questions, sort of differently.
- Facilitator interview, site 6

All staff who were interviewed were asked about how they manage group conversations in which more carers were participating than people with dementia. Some staff members discussed how it was difficult to plan how to balance these conversations because they do not know the participants' needs and wants as they often do not know them well or sometimes meet them for the first time at the session.

Afterwards, [facilitator] talked about how it's difficult to prepare sometimes because you have to adapt what you're going to say depending on who's there and how many's there. Also said she wasn't sure when [person with dementia] was talking when to bring her comments into the group and when she wanted to talk more to herself. Said one time she did [try to bring her in], [her carer] said "oh it's okay, you carry on".
- Fieldnotes, site 1

[Facilitator] said at times he wasn't sure whether [person with dementia] was trying to say something or not, so wasn't sure how much space to give her without it being a silence with everyone looking at her. He said a few times he gave more space and other times less, but sometimes felt uncomfortable when there was more silence and she didn't seem to be wanting to talk after all.
- Fieldnotes, site 2

But staff members described a number of different facilitation strategies to include people with dementia more in group discussions, such as putting more attention on the person with dementia, occupying the carer with something else, or asking the person with dementia more direct questions.

I think I would actually put my attention a bit more on the person with dementia, spending a bit more time with them. I might say something, or I might kind of do…I think maybe it's a bit of a kind of distraction thing, and kind of moving to a slightly different angle on the activity or whatever it is you're talking about. And almost maybe kind of model something a bit, kind of suggest a way of "oh we could do it like this" or "we could answer it this way" or "what do you think if…". Yeah, so I might suggest, I might suggest another option. Or I might be a bit more subtle in my kind of body language towards the person I thought wasn't quite getting the space, I might be asking them quite specific questions directly.
- Manager-facilitator interview, site 2

However, staff also said it was not just about balancing between those who have dementia and those who do not, but also balancing between those with more or less museum experience, or differing levels of interest in the museum:

> *I'm sure that, for example, the guy that was quite excluded, the very middle-class guy who was quite well-educated, and the other lady that's middle class and quite well-educated, they would have quite liked a dementia group but of their own intellectual level.*
> *- Support staff interview, site 2*

They also said it was about balancing between people with dementia who have differing symptoms and a range of abilities, for example between those who are non-verbal and those who are verbally very articulate:

> *The staff discussed the session having a mix of abilities – from James [person with dementia] who was quite articulate and very informed about the art/museum, to Bill [person with dementia] who was non-verbal. Staff said this was the widest range of abilities they'd had on the programme, but felt it still worked well for everyone. They noted the smaller group than expected, which meant that everyone got individualised attention as there was a high staff to participant ratio.*
> *- Fieldnotes, site 2*

Staff interviews at sites 1 and 2, and a session observation at site 4, suggested the mechanism was not just about poor facilitation, however. They suggested that the session being a new, enjoyable activity for the carer, which they do not have very often, meant that carers dominated conversations through their enthusiasm. This may also link with the questions around who the programme is for from the perspective of the dyad. For example, for one dyad at site 1, the sessions seemed more for the carer's interest than the person with dementia's, and the carer was usually very often involved in group conversations in the session whereas the person with dementia was rarely involved unless asked a direct question.

> *After the session, the facilitator said she notices the support workers can ask for lots of historical facts, which isn't what the session is about and can overload people.*
> *- Fieldnotes, site 4*

> *I think one of the dangers sometimes then is [the carers] get really comfortable and also really enjoy it and sometimes they dominate just by the amount of time that they then want to speak, because it's also a space that they're not getting very much.*
> *- Manager interview, site 1*

There was no evidence directly related to the theory's outcome of whether the person with dementia then cannot engage in the session. The only two people with dementia who were interviewed did participate in group conversations. However, there was evidence around considering what 'engagement' means. In fieldnotes at sites 1, 2, and 3, it was noted that some people with dementia seemed to be engaged non-verbally – through smiling, nodding, and pointing, for example. It is possible that, in group conversations, some people with dementia are engaged in the way they want to be, or that works for them, but this is non-verbal rather than verbal, whereas the carers are engaged in a verbal way. For example, the facilitator at site 2 quoted earlier, who was concerned whether he was giving a mostly non-verbal person with dementia the right amount of space to speak, also said:

[Facilitator] said that [person with dementia] was mostly non-verbal, and he felt it was nice that she was included in a lively conversation, even if she couldn't contribute verbally herself. He said she seemed very engaged with the painting and smiling at people.
- Fieldnotes, site 2

Allowing for different ways of engaging may mean carers dominating conversations is not preventing all people with dementia from engaging, if engagement means something more non-verbal to them. It was not possible to determine what 'engagement' meant to individuals in the sessions I attended as I often only saw them in that one session, and not in any other situation or setting to see if they responded differently, for example, to a different type of facilitation. This means it is not possible to determine whether they were being prevented from engaging in the way they wanted.

This theory has therefore been refined. As this research concerns the impact of including carers, the issues around needing to balance between different members of the group for other reasons, such as familiarity with museums, has not been included in the theory.

24. Where there is poor facilitation or the carers are particularly enthusiastic about the activity (M/res), and the person with dementia finds it more difficult to speak up in a group setting (C), carers dominate group conversations (M/res), so the person with dementia cannot engage verbally, does not have a sense of belonging in the group, and cannot gain potential positive outcomes (O).

Theory 25 – (review theory n)

From the review:

"*Where carers are not in contact, or have limited contact, with other carers (C)*, the activity happening in a group means carers meet others in similar situations (M/res) and feel connected to others in a similar situation and the wider community (M/rea), so feel less socially isolated (O), and leads some to continue friendships outside the group (O) or join other dementia-friendly groups (O)."

The context for this theory was only hypothesised in the review, rather than developed through direct evidence within the review papers. There was some evidence to support it in this evaluation, where the 'shrinking social world' means the family carer has limited contact with other carers, in interviews with staff at sites 2 and 6, and one interview with a carer at site 4.

…because it's hard work and can be quite isolating because people do slip away from friendships because they can't quite handle the fact that that person, the other person, has dementia. I witnessed this with a friend who lost her husband recently and she's my age and she was dropped beca…particularly by her husband's male friends, they just, they slipped away. Her girlfriends rallied around. And it was hard work for her. No break, that was partly because she was committed to being the carer, and it's jolly hard work.
- Facilitator interview, site 6

The same carer from site 4 and two members of staff at sites 3 and 4 suggested that carers feel less isolated through meeting others in a similar situation in the group (the theory's mechanism resource).

But I do think in the session that a lot of people find quite a comfort in meeting other people who are also caring for someone with dementia so that's quite a nice addition.
- Manager interview, site 4

And it also helped me seeing people with similar problems and, you know, realising that you're not alone.
- Carer interview, site 4

Staff interviews at sites 4 and 6, and session observations at sites 3 and 5, suggested that carers also feel less isolated through sharing information, such as other groups they might find interesting.

[The carer] also spoke to the [carer] who came alone and at the end told her about some volunteering she did near to where the person who came alone lived, and that she thought she would really enjoy it if she came too.
- Fieldnotes, site 5

The one family carer who was interviewed said she then joined other dementia-friendly groups as a result of coming to the museum sessions:

In the beginning we weren't going out very much and [the museum programme] was a place to go, for him to go. And it encouraged me to get us out more and do more things.
No, no, I was really sort of cautious about going out, then I realised it was really sort of easy and he was fine so, so we did it more.
- Carer interview, site 4

But neither she nor her husband said they continued friendships outside of the sessions. Four studies in the review did report some, though not all, planning to socialise outside the group or continuing friendships (Baker, 2014; Burnside et al., 2017; Lamar, 2015; Roe et al., 2016). This aspect of the outcome will therefore remain part of the theory, but the word "some" will also remain to indicate the difference between this outcome and the outcome of feeling less socially isolated which can be applied more widely.

And although I see the same people often, besides coming here I don't sort of socialise with them at all, it just hasn't happened that way. But they're familiar, you know, so it's just nice to see them and say hello and see how they're getting on.
- Carer interview, site 4

Staff members from sites 1, 2, and 4 suggested that repeated visits or having a regular group of attendees helped sessions to be more social, as people were able to get to know each other over time which meant they formed closer relationships and were then more supportive of each other. This will not be included in the wording of the theory, but will be considered in the conclusions and implications drawn, particularly in terms of why some carers may socialise afterwards but others do not.

When we did have a bigger group and there were people like Adam, like the other guy who was a musician, there was Bill and Bill's wife, and quite a big group, they were turning up and talking to each other more, so we have to do that, less of the we're making chit-chat when everybody arrives, they were sort of saying "oh how are you, what have you been doing?". And it was more social for them.
- Facilitator interview, site 1

Observations of sessions at sites 1, 2, 3, and 6, and staff interviews at sites 1 and 2 suggested carers also socialise in the session with staff, not just other members of the group. This may link with the 'wider community' aspect of theory (n).

During the tea and cake, [dyad 1 carer] talked to [facilitator] about the exhibition…[Volunteer] sat next to [dyad 4 person with dementia] and they talked to each other about both having recently moved to the area.
- Fieldnotes, site 1

Theory (n) has not been altered as a result of any results from the evaluation.

25. Where carers are not in contact, or have limited contact, with other carers (C), the activity happening in a group means carers meet others in similar situations (M/res) and feel connected to others in a similar situation and the wider community (M/rea), so feel less socially isolated (O), and leads some to continue friendships outside the group (O) or join other dementia-friendly groups (O).

Theories 26 & 27 – (review theory o)

From the review:

> "A positive experience in the museum (M/res), in the context of a 'shrinking world' for the dyad (C), makes the dyad feel the museum is a 'safe place' they can return to (M/rea), so they intend to return outside of the group (O) and feel less socially isolated (O)."

The studies in the review which contributed to the development of this theory all described participants *intending* to return to the museum, rather than having any evidence they actually did. As well as providing further evidence of dyads intending to return, this evaluation found some dyads actually did return.

Both dyads who were interviewed said they used to go to the museum prior to dementia, often when they were younger, but, since coming to the programme, now come regularly outside of sessions. One staff member at site 3 also described meeting a person with dementia and her family who had returned to the museum outside of the session.

But that one lady [with dementia] who had the bottle [in an object-handling session], she held the bottle for ages and there was a second bottle that was similar, and then the next month she didn't come, but I saw her in the galleries, in the [gallery]. And I said "Oh it's really nice to see you again. Who are you here with today?" And she said "Oh I'm here with my family, it's my birthday".
- Manager-facilitator interview, site 3

Yeah every once in a while we would come [to the museum]. Not very often, but we would bring the kids here or if we had a visitor we would bring them. But we come a lot more now [since coming to the sessions].
- Carer interview, site 4

One staff member at site 1 said carers would tell her they would come outside of the session to use the café as a place to meet, suggesting the museum had become a safe place to return to:

We've had couples who've started coming when the person with dementia is not so severe and then as time goes on, and I think people's social circles often get smaller and people get, both people can get more timid about going out or being in new spaces. And people have said that because they've carried, they've started going to the museum programme, at a certain point that's still a space that they can go out. And say if they're going into town that they can meet friends at the museum café, for example.
- Manager interview, site 1

As illustrated by the previous quote, there was some suggestion that the museum becoming a 'safe place' becomes more important as a person's dementia progresses and the 'shrinking world' of the dyad increases. Where the dyad have had experience of the museum in sessions when the person with dementia's dementia was more mild, it is a familiar place they could return to, and a place in which the carer already has knowledge of the space, logistical access, and level of support. One of the dyads tracked at site 1, who seemed to be in a transition of needing more support and increasingly struggling to go out, was also mentioned in a staff interview who suggested the museum had become one of very few 'safe places' for them:

> *…and now I think, I think he's the one that pushes them to come. I think, one, because he enjoys it, and also because there's fewer and fewer things he can persuade his wife to do and that they can get to….So I do feel like that's quite a social lifeline for that couple now.*
> *- Manager interview, site 1*

A staff member at site 3 also described examples of care home staff returning outside of a session with other residents, opening up the museum to people with dementia who had not attended the programme themselves, and suggesting it was perceived as a safe and interesting place to go with the people with dementia they support.

> *Because one of the care homes, I saw a few months later, they came back [outside the session] but with different people.*
> *- Manager-facilitator interview, site 3*

A staff member at site 2 said professional carers reported they intended to return to the museum outside of their caring role, such as visiting with family members. However, she, and all staff members, acknowledged it was difficult to get evidence of whether people do actually return. As most museums, and all museums in this research, do not require anyone to sign in, there is no record of who has visited the museum when. Staff members therefore only know if someone has returned to the museum if they tell them or they happen to be working on the same day and see them, as per the manager of site 3 quoted above.

The sessions may also open up the museum for professional carers in particular. Professional carers who work at a residential home, for example, do not necessarily choose to come to the museum sessions, but attend as part of their job role. There was some evidence from descriptions in staff interviews at sites 2, 3, and 6 of professional carers who were disinterested in the museum, but then enjoy the session, and return either with other residents or outside of their caring role.

> *[Volunteer] said when she greeted the group from the care home she thought "this is going to be a hard sell". She said before they came in they stood outside and the carers had a cigarette, and she heard them saying they wished they could stay outside in the sunshine and weren't looking forward to the visit. When they came in, [volunteer] said she tried to enthuse about things they were passing on the way the lift but the group were "completely silent and didn't respond". But said she was pleasantly surprised in the way the carers totally opened up once they got settled into the session. She said that every time they run this sort of programme there are always one or two who she gets that feeling with but then they always leave feeling energetic and full of enthusiasm.*
> *- Fieldnotes, site 2*

This theory also links with the ideas around the carer controlling access to the sessions in Theory 4. If carers control the access for the dyad, then the carer feeling comfortable in the space not only helps them both to attend the session initially, but the feeling that it is a 'safe place' is also the mechanism

which leads them to return outside of the session. If the person with dementia attended the group alone, it would be unlikely to become a safe place for the carer, and so unlikely the dyad would go to the museum outside of sessions.

Theory (o) has not been revised in the light of evidence from the evaluation, but a new theory has been developed specifically related to disinterested professional carers:

26. A positive experience in the museum (M/res), in the context of a 'shrinking world' for the dyad (C), makes the dyad feel the museum is a 'safe place' they can return to (M/rea), so they intend to return outside of the group (O) and feel less socially isolated (O).

27. Professional carers who would not choose to come to the museum and are not interested in it (C), experience an engaging, participatory session where carers are included (M/res), so feel the museum is an enjoyable or interesting place (M/rea), and return outside of the session (O). They return in two ways:
   a) Personally outside of work roles (such as with family)
   b) Professionally with other residents who did not attend the session (the museum becomes perceived as a good place to visit with the people with dementia they support more generally, and the sessions indirectly open up the museum to people with dementia who did not attend a session themselves).

Theory 28 – (review theory p)

From the review:

> "A positive experience in the museum (M/res), in the context of a 'shrinking world' but where the carer does not have capacity to include leisure in day-to-day life (C), means the carer does not see the museum as a place they can return to (M/rea), so they do not intend to return and do not feel less isolated (O)."

Museum staff at sites 1, 4, and 6 described instances where carers (and so dyads) had not returned to the museum due to a lack of capacity or resources to do so:

> *[Support staff] also said that as [the dyad] left to get into the taxi, [carer] gave her a big hug and said "thank you so much for this". She said in the session [carer] had said [person with dementia] doesn't get to go out very often as her family can't pay for wheelchair-accessible taxis, so she usually only goes out once a month, so that's why she's so happy being here. [Contextual note: site 2 pays for participants' travel to the sessions]*
> *- Fieldnotes, site 2*

Although carers always have some responsibility for the person with dementia, the programme also always takes some responsibility from carers in terms of the structure of the session, logistical support within the museum, and the activity and its clean-up if required. Going to the museum outside of sessions means going to the museum without this additional support, which may mean the level of caring responsibilities is too much for the carer. One staff member at site 4 also pointed out that many museums do not have a quiet rest area, so dyads could not take a break if needed during a trip, whereas the tea and coffee room can be used for this purpose during a session.

> *I think also I can imagine organising a visit to a museum that's outside of that kind of structured format where you're kind of looked after by a group of people must be quite stressful and kind of depends on how people are feeling that day and it's, I guess there must be a bit of a sense of "okay, we can come here and then we're going to get told where to go*

and what to look at". Because it's quite an overwhelming building and there's lots of stuff in here, and you can get lost very easily.
- Manager interview, site 6

The theory from the review was therefore revised as follows:

28. A positive experience in the museum (M/Res), in the context of a 'shrinking world' but where the carer does not have capacity to include leisure in day-to-day life or who need the additional logistical support of a session (C), means the carer does not see the museum as a place they can return to outside of sessions (M/rea), so they do not intend to return and do not feel less isolated (O).

References

Baker, E. L. (2014). *Arts Interventions in Dementia Care*. Canterbury Christ Church University.

Blandin, K., & Pepin, R. (2017). Dementia grief: A theoretical model of a unique grief experience. *Dementia*, *16*(1), 67–78. https://doi.org/10.1177/1471301215581081

Burnside, L. D., Knecht, M. J., Hopley, E. K., & Logsdon, R. G. (2017). *here:now* – Conceptual model of the impact of an experiential arts program on persons with dementia and their care partners. *Dementia*, *16*(1), 29–45. https://doi.org/10.1177/1471301215577220

Camic, P. M., Baker, E. L., & Tischler, V. (2016). Theorizing how art gallery interventions impact people with dementia and their caregivers. *Gerontologist*, *56*(6), 1033–1041. https://doi.org/10.1093/geront/gnv063

Camic, P. M., Tischler, V., & Pearman, C. H. (2014). Viewing and making art together: A multi-session art-gallery-based intervention for people with dementia and their carers. *Aging and Mental Health*, *18*(2), 161–168. https://doi.org/10.1080/13607863.2013.818101

Chappell, N. L., Reid, R. C., & Dow, E. (2001). Respite reconsidered: A typology of meanings based on the caregiver's point of view. *Journal of Aging Studies*, *15*(2), 201–216. https://doi.org/10.1016/S0890-4065(00)00026-8

Dalkin, S. M., Greenhalgh, J., Jones, D., Cunningham, B., & Lhussier, M. (2015). What's in a mechanism? Development of a key concept in realist evaluation. *Implementation Science*, *10*(1), 49. https://doi.org/10.1186/s13012-015-0237-x

Duggan, S., Blackman, T., Martyr, A., & Van Schaik, P. (2008). The impact of early dementia on outdoor life: A "shrinking world"? *Dementia*, *7*(2), 191–204. https://doi.org/10.1177/1471301208091158

Flatt, J. D., Liptak, A., Oakley, M. A., Gogan, J., Varner, T., & Lingler, J. H. (2015). Subjective Experiences of an Art Museum Engagement Activity for Persons With Early-Stage Alzheimer's Disease and Their Family Caregivers. *American Journal of Alzheimer's Disease & Other Dementiasr*, *30*(4), 380–389. https://doi.org/10.1177/1533317514549953

Humphrey, J., Montemuro, M., Coker, E., Kilgour-Walsh, L., Moros, K., Murray, C., & Stanners, S. (2017). Artful Moments: A framework for successful engagement in an arts-based programme for persons in the middle to late stages of dementia. *Dementia*. https://doi.org/10.1177/1471301217744025

Jagosh, J. (2017). The "Context + Mechanism" Association: Mastering a Key Heuristic in Realist Evaluation for Innovating Complex Programmes and Policy. Retrieved September 24, 2020, from https://www.youtube.com/watch?time_continue=4&v=e5CgJJwzayI&feature=emb_logo

Johnson, J., Culverwell, A., Hulbert, S., Robertson, M., & Camic, P. M. (2017). Museum activities in dementia care: Using visual analog scales to measure subjective wellbeing. *Dementia*, *16*(5), 591–610. https://doi.org/10.1177/1471301215611763

Kinsey, D., Lang, I., Orr, N., Anderson, R., & Parker, D. (2019). The impact of including carers in museum programmes for people with dementia: a realist review. *Arts and Health*, *00*(00), 1–19. https://doi.org/10.1080/17533015.2019.1700536

Klimova, B., & Kuca, K. (2016). Speech and language impairments in dementia. *Journal of Applied Biomedicine*, *14*(2), 97–103. https://doi.org/10.1016/j.jab.2016.02.002

Lamar, K. L. (2015). *Impacts of Art Museum-Based Dementia Programming on the Participating Care Partners*. University of Washington.

McGuigan, K. A., Legget, J. A., & Horsburgh, M. (2015). Visiting the museum together: Evaluating a programme at Auckland Museum for people living with dementia and their carers. *Arts & Health*, *7*(3), 261–270. https://doi.org/10.1080/17533015.2015.1045531

Roe, B., McCormick, S., Lucas, T., Gallagher, W., Winn, A., & Elkin, S. (2016). Coffee, Cake & Culture: Evaluation of an art for health programme for older people in the community. *Dementia*, *15*(4), 539–559. https://doi.org/10.1177/1471301214528927

Scholl, J. M., & Sabat, S. R. (2008). Stereotypes, stereotype threat and ageing: Implications for the understanding and treatment of people with Alzheimer's disease. *Ageing and Society*, *28*(1), 103–130. https://doi.org/10.1017/S0144686X07006241

Seignourel, P. J., Kunik, M. E., Snow, L., Wilson, N., & Stanley, M. (2008). Anxiety in dementia: A critical review. *Clinical Psychology Review*, *28*(7), 1071–1082. https://doi.org/10.1016/j.cpr.2008.02.008
