# Supplementary material for: A conceptual model of the impact of including carers in museum programmes for people with dementia
Source: Dementia (London). 2022 Sep 22;21(8):2584–600. doi: 10.1177/14713012221126803 (PMC9583285; doi:10.1177/14713012221126803)
Supplement: Supplemental Material - A conceptual model of the impact of including carers in museum programmes for people with dementia [file sj-pdf-3-dem-10.1177_14713012221126803.pdf]

Supplementary File 3. Programme theories mapped to core processes

Numbered programme theories can be found in Supplementary File 1.

Core process	Programme theory number
Caring responsibility	
Carers hold the ultimate responsibility for the person with dementia (so the museum does not have to).	2
The carer's level of responsibilities in the group can prevent them from engaging in and enjoying the activity	2, 20, 23
High staffing levels and the session structure take some responsibilities from the carer	1, 2
Serving tea to carers helps them to feel the session is also for them, to socialise, and to relax after a stressful journey.	8
The carer can enable the person with dementia to participate	2, 13, 22
Carers bring knowledge of the person with dementia which helps the museum staff to support the person with dementia.	22
Museum staff can have differing expectations of different carer types, impacting the carer's experiences	20, 23
Session function	
Some dyads enjoy the session together, but others use it as an opportunity for traditional respite from one another	1, 2, 3
The dyad can experience shared respite from dementia and their caring roles	14, 15
There can be mismatch in the way the carer and the person with dementia wants to use the session, which can cause tension or distress	2, 3, 13
Controlling access	
Carers control whether the dyad (and so person with dementia) attends and/or returns	4, 26, 27, 28
Preventing engagement	
Carers can prevent the person with dementia from engaging fully	5, 21, 23, 24
Carers can dominate the activities or discussions	24
Comparisons & losses	
The session can highlight losses for the family carer	16, 17, 18, 19
The family carer can compare the person with dementia to other people with dementia in the group or with their past selves	5, 16, 19
Long-term impact of in-the-moment activities	
The impact of the programme can extend beyond the session or with other people with dementia who did not attend	6, 7
The session can build the relationship between the carer and the person with dementia	7, 10, 11, 12, 14, 15
The session gives the dyad new things to talk about outside of caring tasks	7, 11, 15
Carers can learn new ways of interacting with the person with dementia and/or new strategies for working with them	6, 9, 12
The carer may reconsider what the person with dementia (or people with dementia generally) are capable of doing, or see the person with dementia in a new way	6, 9, 11
Reducing social isolation & opening up the museum	
Carers can meet others in similar situations and feel less isolated	25
The museum becomes a safe place for the dyad to return to in the future	26, 28
The museum is opened up as an enjoyable and interesting place to visit for previously disinterested professional carers	27
